# Diagnosis and treatment of cardiac amyloidosis: position statement of the German Cardiac Society (DGK)

**DOI:** 10.1007/s00392-020-01799-3

**Published:** 2021-01-18

**Authors:** A. Yilmaz, J. Bauersachs, F. Bengel, R. Büchel, I. Kindermann, K. Klingel, F. Knebel, B. Meder, C. Morbach, E. Nagel, E. Schulze-Bahr, F. aus dem Siepen, N. Frey

**Affiliations:** 1 Sektion für Herzbildgebung, Klinik für Kardiologie, Universitätsklinikum Münster, Von-Esmarch-Str. 48, 48149 Münster, Germany; 2 Klinik für Kardiologie und Angiologie, Medizinische Hochschule Hannover, Hannover, Germany; 3 Klinik für Nuklearmedizin, Medizinische Hochschule Hannover, Hannover, Germany; 4 Klinik für Nuklearmedizin, Universitätsspital Zürich, Zurich, Switzerland; 5 Klinik für Innere Medizin III (Kardiologie, Angiologie und Internistische Intensivmedizin), Universitätsklinikum des Saarlandes und Medizinische Fakultät der Universität des Saarlandes, Homburg, Germany; 6 Institut für Pathologie und Neuropathologie, Universität Tübingen, Tübingen, Germany; 7 Medizinische Klinik m.S. Kardiologie und Angiologie, Charite Universitätsmedizin Berlin Campus Mitte, Berlin, Germany; 8 Klinik für Innere Medizin III, Universitätsklinikum Heidelberg, Heidelberg, Germany; 9 Interdisziplinäres Amyloidosezentrum Nordbayern, Deutsches Zentrum für Herzinsuffizienz, Medizinische Klinik I der Universität Würzburg, Würzburg, Germany; 10 Institut für Experimentelle und translationale kardiovaskuläre Bildgebung, Universitätsklinikum Frankfurt, Frankfurt, Germany; 11 Institut für Genetik von Herzerkrankungen (IfGH), Universitätsklinikum Münster, Münster, Germany; 12 Klinik für Innere Medizin III, Schwerpunkt Kardiologie und Angiologie, Universitätsklinikum Schleswig-Holstein, Kiel, Germany; 13 Kommission für Klinische Kardiovaskuläre Medizin, Deutsche Gesellschaft für Kardiologie, Düsseldorf, Germany

**Keywords:** Amyloidosis, Myocardium, Magnetic resonance imaging, Scintigraphy, Endomyocardial biopsy

## Abstract

Systemic forms of amyloidosis affecting the heart are mostly light-chain (AL) and transthyretin (ATTR) amyloidoses. The latter is caused by deposition of misfolded transthyretin, either in wild-type (ATTRwt) or mutant (ATTRv) conformation. For diagnostics, specific serum biomarkers and modern non-invasive imaging techniques, such as cardiovascular magnetic resonance imaging (CMR) and scintigraphic methods, are available today. These imaging techniques do not only complement conventional echocardiography, but also allow for accurate assessment of the extent of cardiac involvement, in addition to diagnosing cardiac amyloidosis. Endomyocardial biopsy still plays a major role in the histopathological diagnosis and subtyping of cardiac amyloidosis. The main objective of the diagnostic algorithm outlined in this position statement is to detect cardiac amyloidosis as reliably and early as possible, to accurately determine its extent, and to reliably identify the underlying subtype of amyloidosis, thereby enabling subsequent targeted treatment.

**Supplementary Information:**

The online version contains supplementary material available at 10.1007/s00392-020-01799-3.

## Preamble

This position statement is the first expert opinion on the diagnosis and treatment of cardiac amyloidosis written by members of the German Cardiac Society (Deutsche Gesellschaft für Kardiologie–Herz- und Kreislaufforschung, DGK).

The information contained in this position statement reflects a systematic review of the current state of knowledge on this issue. In addition, based on the evaluation of current clinical studies and the expertise of the authors involved, clinical recommendations are formulated to sensitize physicians to this disease and, if necessary, to provide support for everyday clinical practice.

Until a few years ago, amyloidosis was still considered a rare multi-organ disease with primarily neurological or hematological manifestation. Consequently, patients were treated primarily by neurologists and hematologists. Due to the heterogeneity of the symptoms, cardiac involvement of this disease was often overlooked or only documented at a late stage of disease—with unfavourable consequences for the prognosis of the affected patients. Meanwhile, it has become evident that “cardiac amyloidosis” is a frequent and, in some patients, the only manifestation, so that early cardiac diagnosis and subsequent cardiac-directed therapy are becoming increasingly important.

In this context, an interdisciplinary cooperation between specialists in neurology, hematology, gastroenterology, pathology, genetics, and cardiology (and other specialist groups) is an indispensable prerequisite for successful care of patients, and the establishment of specialized and interdisciplinary amyloidosis centres—with central involvement of cardiology—is highly useful.

While promising therapies for the treatment of the neurological manifestation were introduced some years ago, specific therapies for the treatment of cardiac amyloidosis have so far hardly been available. However, the targeted treatment of cardiac amyloidosis is increasingly the focus of clinical trials, and in addition to an extension of the label for already available drugs, the approval of new drugs is expected in the near future.

## Methodology of this position statement

The PubMed database was searched for literature published until 08/2020. Scientific publications in German or English language were searched for the keywords “amyloidosis” and “cardiac amyloidosis”, and evaluated for the assessment of the position statement.

It must be noted that prospective and randomized studies on cardiac amyloidosis with a reliable case number—without pharmaceutical sponsorship—are hardly available. It must also be emphasized that some retrospective (partly multi-centre) studies with quite a large number of cases, which have been published in renowned journals and are often cited, have relevant methodological weaknesses. In this respect, a critical assessment of the available data and careful recommendations for the diagnosis and therapy of amyloidosis were necessary when preparing this position statement.

## Background

### Definition and classification of amyloidoses

“Amyloidosis” describes a disease entity caused by the pathologic deposition of misfolded proteins. In these proteins, instead of an alpha-helix conformation, there is a protein misfolding into a beta-leaf structure and the formation of insoluble fibrils, which are deposited in the interstitium of various organs. Destruction of the physiological tissue structure and direct toxicity of the amyloidogenic substrates lead to consecutive organ dysfunction [1].

Amyloidosis affects both sexes and individuals of any age. As a multi-organ disease presenting with a variety of rather unspecific symptoms of different severity, the diagnosis of amyloidosis is often delayed, and the number of undiagnosed cases is probably high.

So far, more than 30 different proteins have been described to cause, when misfolded, amyloidosis. There are localized forms (such as “alpha-atrial natriuretic factor” [AANF]-amyloidosis with deposition of atrial natriuretic peptide in the atria) and systemic forms such as AA amyloidosis in patients with chronic inflammation and deposition of serum amyloid A (SAA). The vast majority of systemic amyloidoses in industrialized countries are attributable to 3 subtypes: the most common form, which accounts for ~ 70% of all amyloidoses, is light-chain (AL) amyloidosis, which occurs due to the deposition of misfolded immunoglobulin light chains mainly in the context of a monoclonal gammopathy or multiple myeloma. Less frequent are the two forms of the so-called ATTR amyloidosis caused by deposition of misfolded transthyretin (either as wild-type [ATTRwt] form or mutated/variant [ATTRv]) [2].

### Amyloidosis with cardiac involvement

In principle, amyloid can be deposited in any organ and in any type of tissue. However, the different forms of amyloid show different organ tropism typical for the respective disease. Systemic forms of amyloidosis affecting the heart, are mainly AL, ATTRwt, and some forms of ATTRv amyloidosis. In the following, we will primarily focus on the diagnosis and therapy of cardiac relevant forms of amyloidosis.

Cardiac amyloidosis is typically associated with pronounced left-ventricular (LV) wall thickening (≥ 15 mm end diastolic), which can, however, be less pronounced in early stages (< 15 mm). The term “hypertrophy” for this form of LV wall thickening is not quite correct, since “hypertrophy” is primarily a histological diagnosis and refers to an increased size of cardiomyocytes, whereas cardiac amyloidosis is characterized by wall thickening due to interstitial amyloid deposits. Nevertheless, the term “hypertrophy” has also become established in the literature for cardiac amyloidosis.

#### Pathophysiology of AL amyloidosis

The underlying cause of AL amyloidosis is usually a small clonal B cell or plasma cell population, whereas only about 10% of patients have overt multiple myeloma or, in rare cases, a secretory active B-cell lymphoma. In approximately 70% of AL amyloidosis cases, lambda light-chain expression can be found [3]. The cardiac dysfunction observed in AL amyloidosis is attributed to the interstitial deposition of the immunoglobulin light chains, but also to their direct toxicity. Amyloidogenic free light chains can induce in myocytes a lysosomal dysfunction, oxidative stress, apoptosis, and dysregulation of MAP kinase signaling transduction pathways and autophagy. These findings suggest that direct intracellular cytotoxic effects of immunoglobulin light chains are at least partially responsible for the rapid progression of the disease and poor prognosis [4].

#### Pathophysiology of ATTRv and ATTRwt amyloidosis

Transthyretin (TTR) is a transport protein for retinol and thyroid hormone synthesized by the liver, which circulates as a stable tetramer. Transthyretin shows a tendency to form amyloid fibrils when the tetramers dissociate into TTR monomers. The holoretinol-binding protein (RBP) binds and stabilizes tetrameric TTR, suggesting that low concentrations of RBP may be a risk factor for ATTR cardiomyopathy. The cleavage of the TTR tetramers into amyloidogenic monomers susceptible to aggregation is therefore regarded as a speed-determining step in amyloid formation [5]. The destabilization of tetramers and the accumulation of TTR monomers are either the consequence of gene mutations in the *transthyretin* (TTR) gene (ATTRv) or, if absent, are associated with age-related processes that are only partially understood (ATTRwt) [6]. It is unclear which factors explain the higher prevalence in men, the preferred accumulation in certain tissues and the later manifestation of ATTRwt, although the protein is present from birth.

To date, more than 100 mutations have been described in the *TTR* gene, although most ATTRv amyloidoses are caused by p.Val142Ile (also known as Val122Ile) and p.Val50Met (also known as Val30Met) mutations. These pathogenic amino acid substitutions induce conformational changes that destabilize the native tetramer structure and increase the likelihood of their dissociation in amyloidogenic TTR monomers. In this context, it is important to mention that, in addition to cardiotoxic TTR amyloid fibrils, non-fibrillary amyloidogenic TTR molecules are likely to have a cardiotoxic potential [7].

#### Pathophysiology of AA amyloidosis

AA amyloidosis is caused by the deposition of AA fibrils, which are formed from a fragment of the serum amyloid A (SAA) protein in the context of long-lasting inflammatory states. In contrast to ATTR amyloidosis, no mutations are known to promote the formation of fibrils. Nevertheless, there seems to be a genetic predisposition to develop AA amyloidosis. While AA amyloidosis mainly manifests in the gastrointestinal system and kidneys, heart involvement is rather rare. Important causes of AA amyloidosis are uncontrolled chronic inflammatory rheumatic diseases (e.g., rheumatoid arthritis), chronic inflammatory bowel diseases (ulcerative colitis, Crohn's disease), chronic infections (e.g., tuberculosis), and hereditary autoinflammatory diseases (e.g., familial Mediterranean fever).

In the liver, affected patients experience an increased production of the SAA protein, C-reactive protein (CRP), and other proteins of the acute phase reaction due to a chronic inflammatory condition under the influence of interleukin(IL)-1, IL-6, and tumor necrosis factor (TNF)-α. SAA is coupled to lipoproteins (HDL_SAA_) and is taken up into the cell by HDL receptors. As a result of limited proteolysis of the SAA, the AA fragment is formed, which leads to the formation of a matrix in which the characteristic insoluble fibrils can form as a result of a change in conformation and interaction with the amyloid-enhancing factor (AEF), serum amyloid P (SAP), glycosaminoglycans, and other membrane proteins [8].

### Epidemiology of cardiac amyloidoses

The exact prevalence and incidence of cardiac amyloidosis is unknown and varies greatly from region to region and between different ethnicities. In USA, a significant increase in the prevalence rate of cardiac amyloidosis among Medicare beneficiaries (8–17 per 100,000 person-years) and incidence rate (18–55 per 100,000 person-years) was observed from 2000 to 2012 [9]. Moreover, approximately 2200 new cases of AL are diagnosed every year. The prevalence of AL amyloidosis has increased significantly (2.6-fold) between 2007 and 2015, from 15.5 cases per million in 2007 to 40.5 in 2015 [10]. Detailed information for Germany is not yet available. AL amyloidosis occurs in about 10% of patients suffering from multiple myeloma. Between 50 and 70% of patients with AL amyloidosis show cardiac involvement [6], which is decisive for morbidity and mortality, since in patients with untreated, advanced cardiac AL amyloidosis and heart failure, the mean survival time is only about 6 months [11]. In AL amyloidosis, modern treatment strategies have made it possible to extend the median overall survival to > 5 years—at least in patients with less advanced stages [12].

ATTRwt amyloidosis predominantly affects the heart and shows a striking male dominance. The prevalence increases significantly with age: up to 25% of patients over 80 years of age show amyloid deposits in histopathological examinations [13]. Recent studies suggest that ATTRwt may be a causal factor in up to 10% of elderly patients with symptoms of heart failure [14]. In an autopsy study, 109 patients with heart failure and preserved ejection fraction (HFpEF) were examined, in which 19% of the patients showed amyloid deposits in the left ventricle, indicating that ATTRwt might play an etiologically important role also in HFpEF [15].

In contrast to ATTRwt, ATTRv amyloidosis is clinically quite variable in its prevalence and organ involvement depending on the specific TTR gene mutation: peripheral polyneuropathies are particularly common in addition to cardiac involvement. One of the most common mutations is the p.Val142Ile mutation, which occurs in 3–4% of the African population, and the p.Val50Met mutation, which is prevalent in northern Portugal and Sweden. So far, no data exist on the TTR mutation frequency in German amyloidosis patients with cardiac involvement. The experience of most of the authors of this position statement suggests that the proportion of ATTRv amyloidoses among all ATTR amyloidoses in Germany is significantly lower than 10% and thus may differ from other European countries.

Detailed information on the frequency of AA amyloidosis is difficult to obtain. In fact, AA amyloidosis is possibly the most common form of amyloidosis worldwide, as it is associated with a variety of chronic inflammatory diseases. Registries report a prevalence of AA amyloidosis in about 10% of all patients with rheumatoid arthritis (RA). In a Finnish autopsy study of deceased RA patients, the frequency was reported to be up to 30% of all RA patients [16]. However, as anti-inflammatory therapeutic options improve, the prevalence of AA amyloidosis is likely to decrease.

## Clinical manifestation

### Clinical and cardiac manifestations of AL amyloidosis

When considering the total population, AL amyloidosis mainly affects the elderly, but may also affect younger patients in rare cases: the mean age at diagnosis is about 60 years. The clinical symptoms depend on the affected organs and the extent of organ involvement.

Non-specific symptoms such as weight loss, fatigue, dyspnoea on exertion, and oedema occur frequently [1]. Macroglossia with indentations in the lateral parts of the tongue, which is pathognomonic for AL amyloidosis and can lead to swallowing difficulties, and even life-threatening respiratory problems as well as a periorbital purpura (which typically occurs after minor injuries or physical exertion) are only detectable in a few patients with AL amyloidosis [17]. The most frequently affected organs are the heart (71–90%), the kidneys (58–75%), the gastrointestinal tract (22%), the nervous system (23%), and the liver (16–30%) [1, 18]. The lungs and spleen can also be affected by amyloid deposits. Involvement of the soft tissue is less frequent (in 10–15% of patients).

Cardiac involvement is characterized by symptoms of heart failure (peripheral oedema, dyspnoea, and pleural effusions) due to an existing diastolic and, in the further course, additionally occurring systolic dysfunction with a cardiac phenotype mostly resembling hypertrophic or restrictive cardiomyopathy. Arrhythmias can cause dizziness, syncope, or sudden cardiac death. Amyloid protein infiltration of the atria contributes to the high prevalence of atrial fibrillation and the increased risk of atrial thrombus formation in this disease [19]. In addition, amyloid can be deposited within and/or around the small arterioles of the heart, which can lead to clinical symptoms of angina pectoris or in some cases to myocardial infarction [20].

In patients with microvascular amyloid infiltration, a decrease in coronary flow reserve was detected by positron emission tomography (PET) [21].

Renal involvement may be demasked by proteinuria (with predominant albuminuria) and nephrotic syndrome with peripheral oedema, anasarca, foaming urine, or uremic signs. If the gastrointestinal tract is involved, symptoms such as cachexia, constipation, and diarrhoea (also alternating), heartburn, nausea, and vomiting may occur. Furthermore, there may be gastrointestinal bleeding. The so-called “shoulder pad sign” is characterized by periarticular amyloid deposits, which look like shoulder pads [1]. Progressive bilateral and symmetrical polyarthropathies of the fingers, wrists, and knees can also be caused by AL amyloidosis [18]. Furthermore, lymph-node enlargement may be detectable.

If the nervous system is affected, mixed sensomotoric peripheral neuropathies (20%) with numbness, paraesthesia, and pain up to paresis as well as autonomic neuropathies (15%) with consecutive orthostatic hypotension will occur. Carpal tunnel syndrome is present in about 25% of patients and can occur years before diagnosis.

Coagulopathies such as a bleeding diathesis (e.g., due to an acquired factor X deficiency) or hypercoagulability can also be detected in patients with AL amyloidosis. Atrial thrombi often appear before the first overt manifestation of atrial fibrillation. In the case of an increased bleeding tendency without a detectable coagulation disorder, vascular involvement should be considered. The symptoms and clinical manifestations described so far may also appear only over the course of the disease [1].

### Disease progression and prognosis of AL amyloidosis

Cardiac amyloidosis is often overlooked and, due to the heterogeneity of the symptoms, the diagnosis is often made late, which in turn has unfavourable consequences for the prognosis of the patients. In a study of almost 500 patients with AL amyloidosis, 37% of the patients were diagnosed after ≥ 1 year after the onset of initial symptoms, and a third of the patients visited 5 doctors before the diagnosis could be confirmed [22]. Further factors influencing the prognosis are the number of affected organs and the extent of organ involvement as well as the response to a specific therapy.

The differentiation of local from systemic AL amyloidosis is essential for prognostic assessment. Local amyloidosis, e.g., with isolated involvement of the skin and nails, the lungs, the eye, and the urogenital tract, has a good prognosis. The conversion of a local to a systemic amyloidosis is rare [1]. Cardiac involvement typically is the limiting factor in the mortality of AL amyloidosis and is the mortality reason in about 75% of patients.

The combination of the 3 biomarkers NT-proBNP, troponin T, and circulating free light chains allows a risk stratification according to the “Mayo Clinic Staging System” ([23]; Table 1). In patients with atrial fibrillation or renal impairment, the modified Mayo Clinic Staging System (differentiating a stage IIIB if NT-proBNP > 8500 ng/ml) performed best [24]. The NT-proBNP concentration also serves as a progression parameter for assessing the response to therapy or the progression of the disease. However, it must be taken into account that NT-proBNP concentration is dependent on kidney function, among others, and can be subject to fluctuations regardless of the cardiac condition if renal failure is present at the same time.

**Table 1:** Risk stratification in cardiac amyloidosis using biomarkers

	Amyloid subtype	NT-proBNP	Troponin T	Difference between free light chains kappa and lambda (= FLC-diff)	eGFR	Explanation of the score
Mayo Clinic Staging System	AL	≥ 1.800 pg/ml	≥ 0.025 ng/ml	≥ 18 mg/dl	–	One point per biomarker and thus a total score of 0 to 3 points, which allows the classification into stages I–IV. The mean survival is 94, 40, 14, and 6 months, depending on the stage
Staging according to Grogan et al.	ATTRwt	> 3.000 pg/ml	> 0.05 ng/ml	–	–	Classification into three stages with 4-year survival rates of 57, 42, and 18%, depending on whether none, one, or both biomarkers exceed the specified levels
Staging according to Gillmore et al.	ATTRwt ATTRv	> 3.000 ng/l	–	–	< 45 ml/min	Classification into three stages according to eGFR and NT-proBNP with mean life expectancies of 69 (both markers normal), 47 (one marker pathological), and 24 months (both markers pathological) in the three groups

The prognosis of AL amyloidosis strongly depends on the disease stage and progression, respectively. In patients with non-advanced AL amyloidosis who respond to therapy with haematological remission, median survival was reported to be over 5 years. However, in patients with advanced disease stages and without treatment, the disease usually leads to death after 6–12 months [18]. Therefore, early diagnosis has a significant impact on the survival rate.

### Clinical and cardiac manifestations of ATTRv and ATTRwt amyloidosis

The phenotype of ATTR amyloidosis and also the age at first manifestation depend on the genotype, i.e., on the particular TTR gene mutation. Some mutations result in both neurological and cardiac symptoms, while others either lead to an almost exclusively neurological appearance, especially at a young age, or rarely to an isolated cardiac disease manifestation [25].

The most common phenotype, particularly in patients with ATTRwt amyloidosis, is the cardiac manifestation. The amyloid deposits can infiltrate all structures of the heart and, in addition to the ventricular and atrial wall, can also affect the conduction system, the heart valves, and the coronaries. Thus, the clinical spectrum of cardiovascular involvement is broad, ranging from asymptomatic courses, the occurrence of dizziness and syncope to the development of restrictive cardiomyopathy and progressive terminal heart failure. The cardiac biomarkers NT-proBNP and troponin T are often elevated, but not as pronounced as in patients with AL amyloidosis [25].

Patients with ATTRwt amyloidosis also show a relatively larger left-ventricular wall thickness compared to patients with other forms of amyloidosis [26]. ATTRwt amyloidosis is characterized by a late onset of the disease (usually after the 7th decade of life) and occurs most frequently in men. The heart is mainly affected in up to 90% of patients. Initially, unspecific symptoms such as fatigue, performance slumps, and exertional dyspnoea appear as early symptoms of the disease [27]. During the course of the disease, the patients then present signs of heart failure (87%) and cardiac arrhythmias (65%) such as atrial fibrillation or AV blocks, but also ventricular arrhythmias [28, 29].

The primary site of extracardiac manifestation is the carpal tunnel. Carpal tunnel syndrome (CTS) occurs in up to 70% of patients with ATTRwt amyloidosis 5–10 years before cardiac manifestation. Furthermore, spinal canal stenosis may occur. An atraumatic biceps tendon rupture occurs in about 33% of patients. Findings such as an existing carpal tunnel syndrome (particularly bilaterally in men), neuropathic pain of unclear origin, orthostatic hypotension, and especially the evidence of unclear left-ventricular wall thickening after the 6th decade of life are indicative for the diagnosis of ATTR amyloidosis [30] **(**Table 2**)**.

**Table 2:** Signs for cardiac amyloidosis

Cardiac amyloidosis should be considered if, in addition to concentric LV wall thickening (≥ 12 mm)—in the absence of hypertensive heart disease—one of the following points is present
Age > 60 years, symptoms of heart failure and still normal-sized ventricles
Low voltage or detection of an AV block in the resting ECG
Evidence of pericardial effusion, interatrial thickening, an echo hyperintense myocardial texture, a wall thickening of the RV, a valve thickening, or an “apical sparing”
Pathognomonic macroglossia with notches in the lateral parts of the tongue
Periorbital purpura (typically after minor injuries)
Atraumatic biceps tendon rupture
Carpal tunnel syndrome (mostly on both sides)
Sensorimotor polyneuropathy, neuropathic pain of unknown origin
Spinal stenosis
Autonomic dysfunction as well as orthostatic hypotension and erectile dysfunction
Vitreous opacity and pathognomonic pupillary changes

In particular, the presence of left-ventricular wall thickening which is not explained by arterial hypertension should always raise the suspicion of ATTRwt amyloidosis in older men [17]. Furthermore, in patients with calcifying aortic stenosis, ATTR amyloidosis was described in up to 32% of male patients aged ≥ 74 [31]. Recent studies suggest a prevalence of 12–16% [32, 33]. The survival of these patients after interventional or surgical valve replacement is significantly worse when compared to patients without amyloidosis [33, 34].

The clinical manifestation of ATTRv amyloidosis depends on the mutation and other factors such as age at diagnosis, heredity pattern, gender, geographical conditions, and ethnicity [28]. The cardiac symptoms in patients with ATTRv amyloidosis are very similar to the symptoms of patients with ATTRwt, but the neurological manifestations can be more pronounced. Axonal sensorimotor polyneuropathy is a classic symptom of ATTRv amyloidosis. It usually begins with a disturbed sensitivity to pain and temperature in the lower extremities with a proximally ascending pattern as the disease progresses. In addition, numbness and tingling as well as muscle weakness and walking disorders may occur. Carpal tunnel syndrome is also very common in patients with ATTRv amyloidosis. Autonomic dysfunctions such as orthostatic hypotension and erectile dysfunction as well as diarrhoea can also occur more frequently. With certain TTR gene mutations, vitreous opacities and pathognomonic pupil changes can also be detected. Involvement of the central nervous system is rare; leptomeningeal amyloidosis has, however, been described in some rare TTR mutations with a neurological clinical picture of dementia, stroke, ataxia, or spastic paralysis.

### Disease progression and prognosis of ATTRv and ATTRwt amyloidosis

Patients with ATTR amyloidosis have a statistically better prognosis compared to patients with AL amyloidosis. However, the natural individual course of ATTR amyloidosis is difficult to assess due to the multiple factors influencing the disease and the heterogeneity of genotypes. In particular, the progression of neurological disease and cardiac manifestation are the leading causes of death in patients with ATTR amyloidosis. Polyneuropathy disability can be classified into three stages: free walking (stage I), dependent on a walking aid (stage II), and wheelchair bound (stage III).

Data on the prognosis of patients with cardiac ATTR amyloidosis depend on the initial individual stage of the disease and the population under consideration. The results of the “Transthyretin Amyloid Outcome Survey” (THAOS), which focused on ATTR patients in the USA, suggest a mortality rate of > 30% within 2 years for patients with ATTRwt, while for ATTRv with the p.Val142Ile mutation, even a mortality rate of > 40% within 2 years was observed [35]. The Transthyretin Amyloid Cohort Study (TRACS) also showed a marked difference in mortality between patients with ATTRwt and ATTRv (p.Val142Ile) (22% vs. 79% after an observation period of ~ 15 months) [36].

In patients with ATTRwt, the cardiac biomarkers NT-proBNP and troponin T can also be used for risk stratification [37]. The staging system of the UK National Amyloidosis Centre provides prognostic information by measurement of the NT-proBNP concentration and the estimated glomerular filtration rate in patients with ATTRv as well as ATTRwt [38] (Table 1). Without treatment, every 6 months, the 6-min walking distance decreased by ~ 26 m, NT-proBNP increased by 1816 pg/ml, and left-ventricular ejection fraction deteriorated by 3.2%. However, the number of patients examined in this study (*n* = 29) was very small. According to a somewhat larger study, the median life expectancy of ATTRwt is 47 months (after diagnosis) and the mortality rate is 64% after 5 years [39].

### Clinical and cardiac manifestation of AA amyloidosis

Since AA amyloidosis primarily affects the kidneys, it usually manifests itself as renal insufficiency, often with proteinuria. At the time of the onset of clinical symptoms, there is usually already a manifest, advanced organ involvement. In earlier stages of the disease, only histological evidence is possible. A gastrointestinal involvement can often also be detected, but this typically remains asymptomatic for a long time. In the course of the disease, diarrhoea, gastrointestinal bleeding, and malabsorption disorders may occur. Liver, spleen, and adrenal gland involvement can often be detected histologically, but does not manifest itself clinically over a long period of time. Cardiac involvement in AA amyloidosis is considered rare and untypical (< 10%) [40, 41]; only one study was able to demonstrate a higher case rate in long-term observation of patients with rheumatological diseases (26%) [42]. Since only case reports on cardiac AA amyloidosis have been available, little is known about clinical and morphological details.

### Disease progression and prognosis of AA amyloidosis

Mean 5-year survival in most studies is about 40%, only in one study, a mean survival time after the initial diagnosis of about 11 years was found [41]. However, it should be pointed out that there have been improvements in the therapy of the respective underlying disease, which might have a positive influence in the future. Predictors of poor outcome include elevated SAA concentration, significantly reduced renal function at the time of initial diagnosis, and cardiac involvement [42]. According to one study, the median survival is ~ 6 years for patients without heart involvement and ~ 2 years for patients with heart involvement [43].

## Diagnostic assessments for cardiac amyloidosis

### Basic laboratory diagnostics

Laboratory diagnostics can help to evaluate organ involvement and its extent in all forms of amyloidosis. Measuring of transaminases, gamma-GT, AP, and bilirubin for the assessment of liver function is oligligatory, and may indicate liver or cardiac involvement with right heart failure and congestion. Total protein and albumin levels should be tested. Since the kidneys are affected in several forms of amyloidosis, creatinine, urea, and the estimated GFR should be determined. The quantification of proteinuria and albuminuria (in particular in 24-h urine collection) is a diagnostic criterion for the presence of renal involvement in AL and AA amyloidosis. White blood cell differential, CRP, electrolytes, and coagulation (INR, PTT) should also be determined in patients with amyloidosis. In AA amyloidosis, SAA is the most important follow-up and activity parameter.

### Specific cardiac biomarkers

The cardiac biomarkers troponin T and NT-proBNP are part of the diagnostic criteria for the presence of cardiac involvement in all forms of amyloidosis. Biomarkers in the normal range virtually exclude relevant cardiac involvement, while elevated biomarkers may indicate cardiac involvement, but are not specific for amyloidosis and must be interpreted in the context of cardiac imaging. Biomarkers are also used for risk stratification in various staging systems (Table 1; [37, 44]).

Single studies have shown a prognostic value for transthyretin (prealbumin) as a serum marker in ATTRwt amyloidosis. Low serum transthyretin levels at the time of initial diagnosis are prognostically unfavourable [45]. For the estimation of the extent of cardiac involvement in AL amyloidosis, the Mayo-classification already mentioned [based on troponin T, NT-proBNP, and difference between the free light chains kappa and lambda (FLC-diff)] is primarily crucial (Table 1**)** [23].

### Specific laboratory diagnostics for suspected AL amyloidosis

If light-chain amyloidosis is suspected, additional laboratory tests are necessary. First, an electrophoresis with determination of the M-gradient and immunofixation electrophoresis in serum and urine are performed, followed by quantitative determination of the free light-chain kappa and lambda in serum as well as the calculation of the ratio and the difference [46]. A kappa–lambda ratio of < 0.26 indicates monoclonal lambda gammopathy, while a kappa–lambda ratio of > 1.65 indicates monoclonal kappa gammopathy. In addition, a quantification of albumin and protein excretion as well as free light-chain excretion should be performed in the 24-h urine collection. A proteinuria of > 500 mg per day (mainly albumin) suggests a renal involvement. The free light chains (including the ratio and difference) are also used to assess the response to therapy.

### Electrocardiography (resting and Holter ECG)

The 12-lead resting ECG is part of every cardiac evaluation in patients with suspected cardiac amyloidosis as well as of every follow-up visit. Typical ECG changes such as low voltage only occur in about half of the patients. The low voltage contrasts with the thickening of the left ventricular wall, which is not due to hypertrophy of the cardiomyocytes, but is caused by amyloid deposits in the extracellular space. In early stages, low voltage is usually not detectable and unspecific. A true low voltage (QRS < 0.5 mV in limb leads and < 1 mV in chest wall leads) occurs in 50% (AL) and 30% (ATTR) of cases of cardiac amyloidosis [47].

Furthermore, cardiac amyloidosis often results in a prolongation of the QTc interval. For example, in patients with left-ventricular wall thickening, a QTc time extension of > 440 ms with a simultaneous Sokolow–Lyon index of < 1.5 mV (according to a smaller study) has a sensitivity of 85% and a specificity of 100% for detecting cardiac amyloidosis [48]. Other ECG signs include pseudo-infarct constellations and QRS broadenings.

In view of modern non-invasive imaging techniques, which allow both a more sensitive and more specific diagnosis of cardiac amyloidosis, the resting ECG plays a subordinate role.

In patients with cardiac amyloidosis, there is a general indication for a Holter ECG. If symptoms such as vertigo, syncope, and palpitations are also present, a (renewed) long-term ECG should be performed. AV blockages and bradycardias are rather common complications of cardiac amyloidosis leading to pacemaker or ICD implantation (depending on the extent of cardiac involvement). In addition to the AV block, the detection of (asymptomatic) atrial fibrillation is also important. Atrial fibrillation has been described in 5–18% of patients with ATTRv and 27–62% with ATTRwt amyloidosis [49] with the indication for oral anticoagulation. The significance of ventricular arrhythmias, on the other hand, is controversially discussed (see "Device therapy for cardiac amyloidosis" section).

### Tilt table test

Tilting table test is rarely performed today and is often unavailable for patients with cardiac amyloidosis. In cardiac amyloidosis, orthostatic hypotension occurs with (secondary) autonomic dysfunction. The tilting table plays a defined role in the clarification of orthostatic dysfunction [50]. As a consequence, antihypertensive drugs and diuretics should be reduced in individual cases. However, a Holter ECG should be carried out first (if necessary also the implantation of an event recorder) to exclude bradycardic or tachycardic arrhythmias.

### Echocardiography

Transthoracic echocardiography is the primary and most widely available cardiological diagnostic imaging tool for patients with suspected cardiac amyloidosis. It is cost-effective, universally available, radiation-free, and has no contraindications. It can also be easily performed as part of a family assessment in ATTRv.

Cardiac amyloidosis often appears in the late stages as restrictive cardiomyopathy with corresponding biatrial dilatation, diastolic dysfunction, and LV wall thickening (usually with an echogenic septal myocardium described as “granular sparkling” phenomenon). In the early stages, wall thickening is often not yet visible. An echocardiographic follow-up should also be carried out regularly (starting 10 years prior to the predicted age of onset and thereafter every year) in asymptomatic individuals who carry the mutation.

In addition to the quantitative description of wall thickening, the assessment of the size of the left atrium (biplane volumetry and indexing to the body surface; LA volume index) and of the diastolic function of the left heart is important. In addition to atrial enlargement, atrial amyloid deposits (thickening of the atrial walls) are also described, which further increase the risk for atrial fibrillation. Besides the transmitral E/A ratio, a tissue Doppler measurement should always be performed in the basal segments of the LV lateral and septal walls. In addition to the E/E′ ratio, the pulmonary venous flow in the right upper pulmonary vein can often be displayed very well. For follow-up (under treatment), these parameters should be recorded.

Cardiac amyloidosis often shows a slight pericardial effusion, which, in most cases, is not hemodynamically relevant. In every echocardiography exam, it should be analysed in the subcostal plane: presence of compression of the right atrium. Cardiac amyloidosis only rarely leads to pericardial tamponade [51]. Complications of cardiac amyloidosis include wall thickening and diastolic dysfunction, as well as mitral regurgitation and post-capillary pulmonary hypertension. In some patients, LV outflow tract obstruction due to increased wall thickness was observed.

Left-ventricular ejection fraction (LVEF) is often only slightly reduced in early stages. In cardiac amyloidosis, measuring LVEF without knowledge of volumes and stroke volume is not a reliable parameter to assess systolic LV function; low-output hemodynamics may be present despite normal LVEF. LVEF is primarily a parameter of radial myocardial function and allows only little information about longitudinal shortening. As the loss in LV longitudinal shortening is compensated by increased radial thickening, the LVEF remains formally “normal” until late in the course of the disease.

Speckle tracking echocardiography (“deformation imaging”) is a newer technique that should be used in all patients with suspected cardiac amyloidosis and is also recommended in current European (ESC) guidelines [52, 53]. The “deformation” is defined as the percentage shortening of a myocardial segment in relation to the initial length. In the 2D image, individual “speckles” (characteristic pixels) are tracked from image to image (“tracking”). From the movement of the “speckles” in a systole, the deformation (i.e., the percentage approximation of adjacent pixels during a systole) can be calculated. In addition to assessing the global strain (“global longitudinal strain” [GLS]), the regional myocardial function can also be assessed with lower intra- and inter-observer variability.

**Fig. 1:**
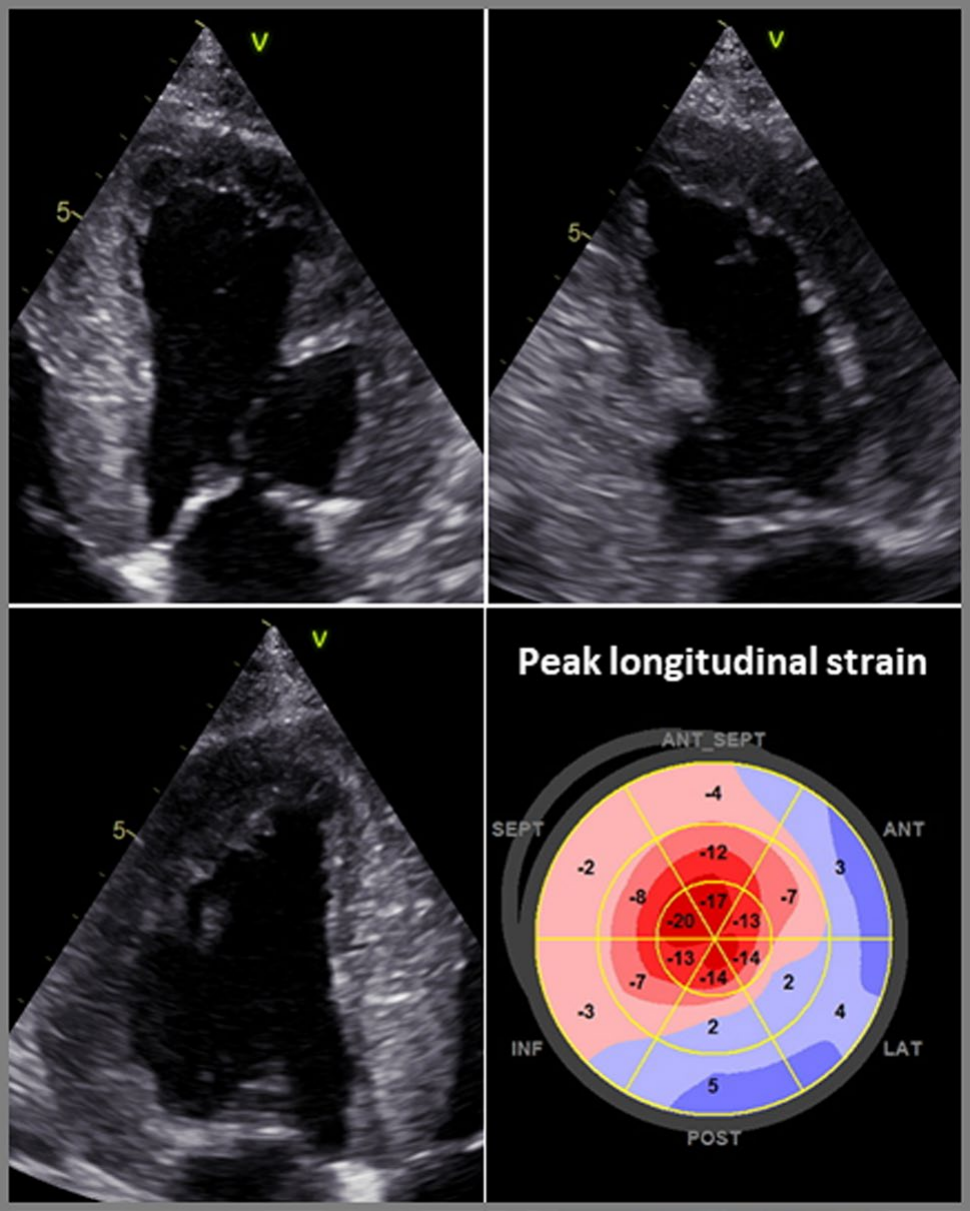
Transthoracic echocardiography with measurement of longitudinal strain and detection of a characteristic “apical sparing” phenomenon in a patient with cardiac amyloidosis

In patients with cardiac amyloidosis, it is usually possible to determine longitudinal systolic function in all myocardial segments (GLS). These patients also typically exhibit the phenomenon of “apical sparing”: this typical pattern shows a disturbance of the longitudinal strain in the basal segments with nearly normal longitudinal function of the apical segments **(**Fig. 1**)**. According to some studies, “apical sparing” is specific and sensitive (sensitivity of 93% and specificity of 82% compared to controls and patients with other forms of LV wall thickening) [54]. It correlates with the scar extent in CMR imaging and is also prognostically significant [55], but cannot reliably distinguish between the different subforms of cardiac amyloidosis [56].

An important differential diagnosis of cardiac amyloidosis is the more common classical hypertrophic cardiomyopathy (HCM)—often caused by sarcomere protein mutations, in which an SAM phenomenon or an intraventricular obstruction is often detectable. Therefore, if the LV wall thickening is unclear, a Valsalva manoeuvre should always be carried out to exclude a dynamic outflow tract obstruction. Other differential diagnoses of amyloidosis include hypertensive heart disease, other storage diseases such as glycogenosis, hemochromatosis, or Fabry disease, which often involves wall thickening with prominent papillary muscles, although papillary muscle involvement can also occur in cardiac amyloidosis. Speckle tracking-based strain imaging can help in the differential diagnosis; the presence of “apical sparing” may indicate cardiac amyloidosis.

Especially, elderly patients with cardiac ATTRwt amyloidosis often have additional cardiac pathologies which have to be clarified by echo. The most important of these is the aortic valve stenosis. In the case of coincidence of cardiac amyloidosis and aortic valve stenosis, often, a “paradoxical low-flow low-gradient” aortic valve stenosis is present, which is often difficult to diagnose if classified only by the pressure gradient. This can only be diagnosed if the continuity method is also used and the stroke volume index is determined.

Cardiac amyloidosis is associated with increased occurrence of intracardiac thrombi. To rule out intraventricular thrombi, in addition to CMR imaging, contrast agent echocardiography is also useful [57]. In addition to transthoracic echocardiography, transoesophageal echocardiography is also an important method before cardioversion or initiation of rhythm control therapy for atrial fibrillation.

### Cardiovascular magnetic resonance imaging

In recent years, cardiovascular magnetic resonance imaging (CMR) has gained considerable importance in the diagnosis of amyloidosis and its distinction from other cardiomyopathies. The following three strengths of the method are particularly important:

- the exact analysis of heart function and anatomy,
- the recording of myocardial infiltration using contrast-enhanced images (“late gadolinium enhancement” [LGE]),
- the recognition of diffuse changes in the myocardial extracellular space with specific mapping techniques.

#### CMR-based analysis of heart anatomy

The recording of global function, wall motion, myocardial mass, wall thickness, atrial size, and the interatrial septum is part of every routine CMR examination. The increased myocardial mass, significant thickening of the myocardium, enlargement of both atria, and thickening of the interatrial septum can be reliably detected in almost all patients. An advantage compared to echocardiography is the somewhat greater accuracy, especially in patients who cannot be examined well sonographically, the better differentiation of trabeculae and papillary muscles, the significantly sharper and higher-contrast epicardial differentiation, and the complete imaging of both atria. Global and regional strain analyses can be performed with, e.g., the feature tracking method using standard CMR (cine) images [58]. Beyond the improved visualization itself, CMR does not offer any diagnostic advantages in terms of merely anatomical visualization or functional evaluation.

#### Detection of infiltration by contrast-enhanced CMR

**Fig. 2:**
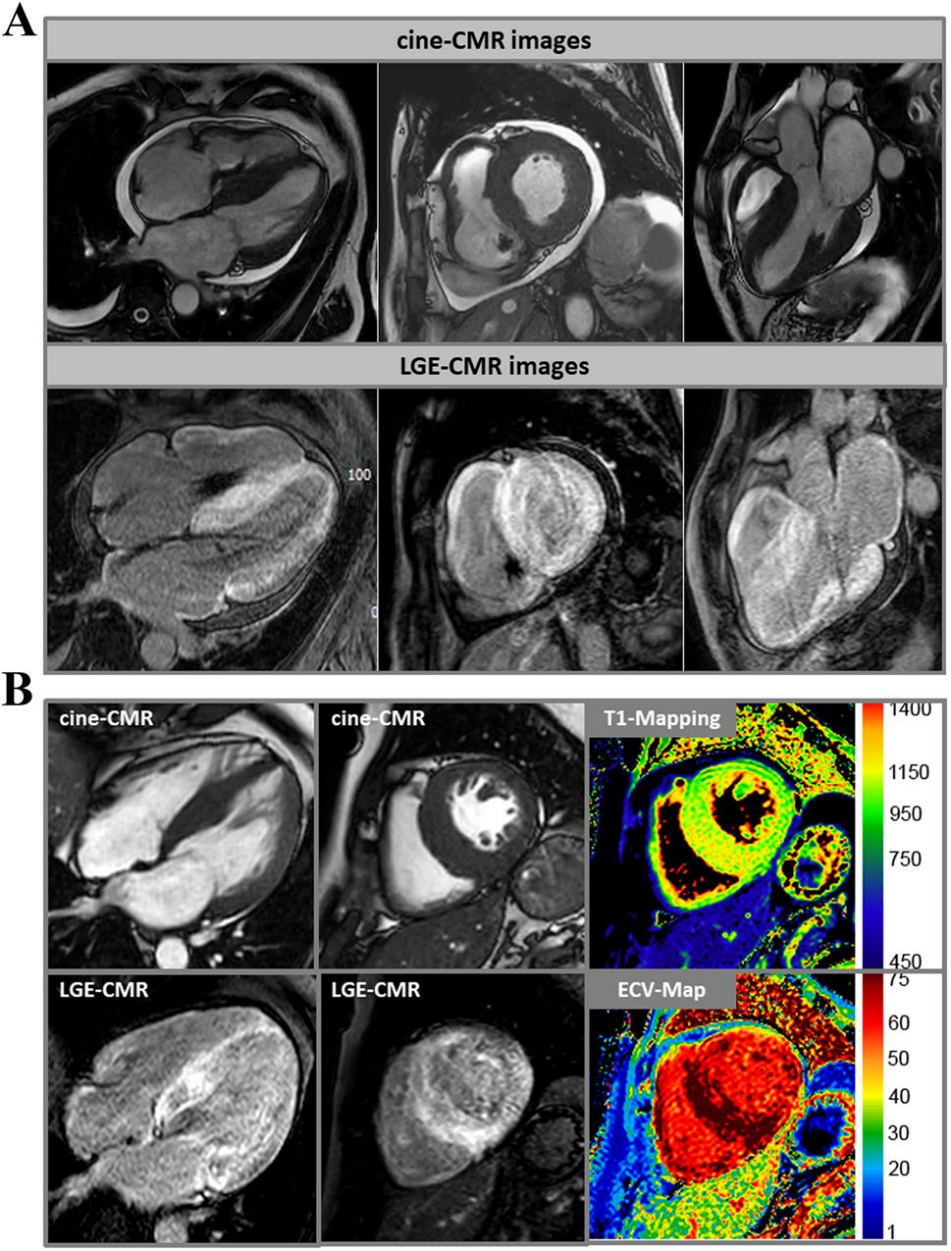
**a** Cardiovascular magnetic resonance (CMR) images of a patient with cardiac amyloidosis and a DDD pacemaker. In the upper row, cine images are shown in a 4-chamber view, in the short axis, and in a 3-chamber view. In the lower series, the corresponding contrast images (“late gadolinium enhancement” [LGE]) are shown with evidence of diffuse LGE in all heart cavities and myocardial sections. **b** In addition to the cine and LGE images, T1 mapping and ECV (extracellular volume fraction) images are shown in the right column. Particularly in the basal septum, measured values of T1 (native) = 1300 ms and ECV = 70% were significantly increased and characteristic for cardiac amyloidosis

The increased interstitial volume in amyloidosis leads to a correspondingly increased volume of distribution for the extracellular gadolinium-containing contrast media (CM) commonly used in CMR [59, 60]. A few minutes after administration of CM, all infiltrated myocardial segments accumulate it and delimit themselves from unaffected or less affected heart segments. The diagnosis is more challenging if the respective myocardial damage is rather diffuse and quite homogeneous, as the LGE method is particularly good at visualizing regional differences, but global changes can appear pseudonormal. An early observation was therefore that, in amyloidosis, the signal intensities in the blood and myocardium rapidly converge, thus eliminating the usual contrast between blood and myocardium. Such a difficulty in achieving the usual contrasts in LGE imaging is a clear indication for the presence of a pronounced amyloidosis. In almost all cases, however, the entire heart muscle is not equally affected, so that regional differences with emphasis on the basal and subendocardial segments can also be recorded with the LGE method. LGE is not restricted to typical vascular territories or sharply defined as in case of myocardial infarction. Often diffuse and in part transmural changes can be seen **(**Fig. 2a**)**. Both the right ventricle and the atrial walls may be affected. These typical and specific LGE patterns allow a clear identification of cardiac amyloidosis with sensitivities of ~ 90% and specificities of ~ 90% [61, 62]. A high-quality CMR study with a characteristic LGE finding as shown in Fig. 2a is diagnostic of cardiac amyloidosis and similar to scintigraphy, the combination of such characteristic LGE findings with negative monoclonal protein studies will result in a high positive predictive value for the diagnosis of ATTR amyloidosis.

With the LGE technique, CMR thus offers a method that allows a direct view into the myocardium and also detects cardiac involvement in which the heart function is not measurably impaired or in which the wall thicknesses are still in normal or otherwise explicable ranges. In addition, the presence of global or transmural LGE is associated with a highly adverse prognosis (HR 2.93–5.4) and goes beyond the prognostic value of other markers (e.g., NT-proBNP or LVEF) [63, 64].

A frequently mentioned problem is the administration of gadolinium-containing CM in cases of impaired renal function. The often held opinion that these CM should not be given in patients with impaired renal function (eGFR < 30) has been relativized in recent years. If cyclic gadolinium chelates are chosen, the administration is usually possible even in cases of impaired renal function, provided that the indication has been strictly defined and the patient has been informed. However, the smallest possible dose (e.g., 0.0075–0.01 mmol/kg body weight) should always be aimed for.

#### The detection of a diffuse change in extracellular space with mapping techniques

With new methods, the magnetization of the heart muscle can be measured directly (“mapping”). Mapping techniques do not require regional differences, but show diffuse abnormalities by changing the absolute values [65]. Native T1 mapping does not require a contrast agent and provides a quantitative measure of the severity of the disease. Native T1 mapping can detect cardiac amyloidosis as accurately as CM-assisted methods and shows significantly higher values for cardiac amyloidosis than in patients with hypertrophic cardiomyopathy, who also show an increase compared to heart-healthy or hypertensive patients [66–68]. Patients with AL amyloidosis usually have more pronounced changes than patients with ATTR amyloidosis (Fig. 2b**)**. The increase in native T1 time for each subtype is correlated with the amount of amyloid deposition. Since T1 standard values may vary depending on the equipment and protocol, each MRI centre should define its own standard values, following the recommendations of the professional societies [69].

For both amyloid subtypes, the amount of native T1 time is closely linked to the prognosis. The significance of possible differences between native T1 time, which indicates changes in intercellular and interstitial space, and extracellular volume (ECV), which is determined by T1 mapping before and after CM administration and has a stronger emphasis on extracellular space, has not yet been conclusively clarified [70, 71]. However, the higher ECV values in ATTR compared to AL indicate pathophysiological differences at the cellular level between different types of amyloidosis. In a direct comparison of the hazard ratio values between ECV, LGE, and bone scintigraphy (based on the use of technetium [^99m^Tc] phosphates), T1 and ECV data were prognostically superior to both LGE and scintigraphy [70]. So far, however, these studies are relatively small monocentric studies with a rather short observation period.

While scintigraphic methods only allow for the diagnosis of amyloidosis, CMR offers the great advantage that different aetiologies associated with LV wall thickening or hypertrophy can be clarified simultaneously and the exact underlying cause can usually be correctly assigned even in the case of a non-amyloidosis-associated LV wall thickening. Therefore, the current heart failure guidelines of the ESC recommend a CMR study for tissue characterization in patients with heart failure symptoms (I/C) [53]. Moreover, CMR is expected to play a central future role regarding non-invasive therapy monitoring in patients with cardiac amyloidosis receiving specific therapies [72, 73] due to its unique capabilities regarding non-invasive myocardial tissue characterization including depiction and quantification of amyloid load.

In summary, CMR offers an indispensable method for the detection of cardiac amyloidosis, both in the context of classical methods (LGE) and by extension to the new mapping procedures, and should be used early in cases of unclear left-ventricular hypertrophy.

### Nuclear imaging techniques

Nuclear imaging approaches for the non-invasive diagnosis of cardiac amyloidosis currently include primarily scintigraphy and positron emission tomography (PET)-based procedures.

#### Scintigraphy for the diagnosis of cardiac amyloidosis

Current scintigraphic methods rely on the use of radionuclides based on ^99m^Tc phosphates. ^99m^Tc phosphates were initially developed for bone scintigraphy in the 1960s and later studied for myocardial infarction imaging [74]. In cardiology, they have experienced a renaissance in recent years in the context of cardiac amyloidosis diagnosis, although the first reports on this topic were already published in the early 1980s [75].

The ^99m^Tc phosphates currently in clinical use for cardiac amyloidosis diagnostics in Europe include ^99m^Tc-DPD (3,3-diphosphono-1,2-propanodicarboxylate) and ^99m^Tc-HMDP (hydroxymethylene-diphosphonate). By contrast, in the United States, ^99m^Tc-PYP (pyrophosphate) is commonly used, while DPD is not approved [76–78]. The exact mechanism that leads to the increased affinity of these radionuclides to amyloid deposits in the heart has not yet been clarified in detail, but is suspected to be due to microcalcifications related to amyloid deposits. Also, the exact reason for the slightly higher affinity of these radionuclides for myocardial ATTR deposits compared to AL deposits is still unclear.

**Fig. 3:**
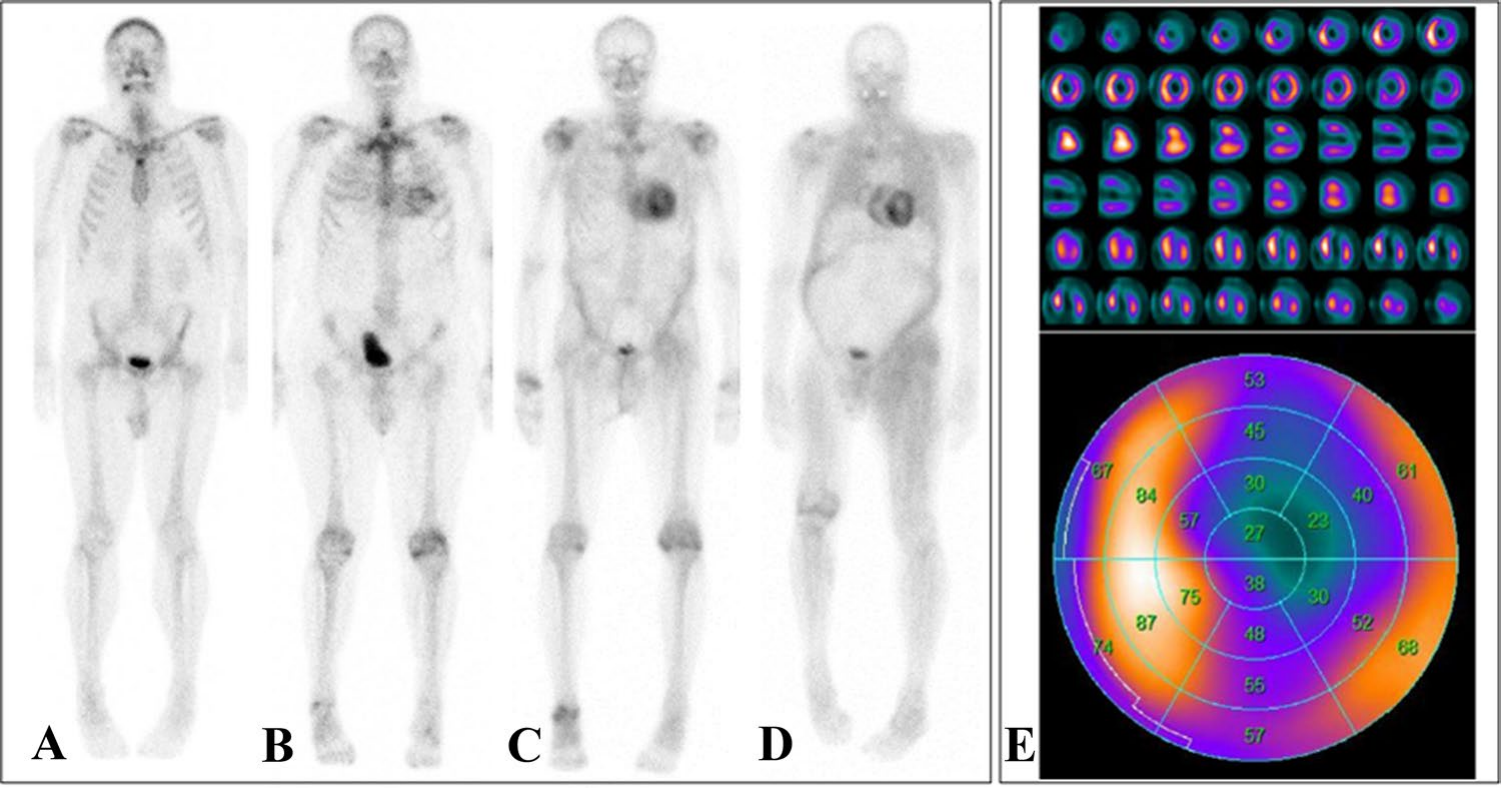
Planar 99mTc-DPD scintigraphies of four different patients with varying degrees of cardiac radionuclide uptake: No cardiac uptake (Perugini score 0, **a**). Light cardiac uptake with preserved delineation of bone tissue (Perugini score 1, **b**). Strong cardiac uptake above that of bone tissue and increased soft-tissue uptake, particularly in the shoulder, abdominal wall, and gluteal region (Perugini score 2, **c**). Strong cardiac and soft-tissue uptake with no discernable bone-tissue uptake, suggesting diffuse amyloid soft-tissue infiltration (Perugini score 3, **d**). In case of myocardial uptake on planar scintigraphy, SPECT imaging should be performed, which allows for a detailed assessment of radionuclide distribution within the left-ventricular myocardium (**e**, short axis, vertical long axis, and horizontal long axis slices at the top and the corresponding polar plot at the bottom; white/yellow indicates high uptake; blue/black indicates little or no uptake)

The applied amount of radionuclides or the whole procedure leads to a low average effective radiation dose of approx. 2–4 mSv per person and examination, which is less than the annual individual background radiation dose from natural sources [79]. Depending on the protocol, the total duration of the examination or stay of the patient from the first injection to the last image acquisition is up to 1–3 h, whereby planar scintigraphy is performed, followed by dedicated cardiac single-photon-emission-computed-tomography (SPECT) imaging in case of positive planar imaging **(**Fig. 3**)**. Of note, no specific patient preparation is warranted. In recent years, the following two approaches in particular have become established for image interpretation: (a) a semi-quantitative visual analysis using the so-called Perugini Score, a point scale of 0 to 3, where 0 = no cardiac uptake and normal bone uptake, 1 = mild cardiac uptake less than bone uptake, 2 = moderate cardiac uptake and relatively equal bone uptake, and 3 = high cardiac uptake and only mild or absent bone uptake [78]; (b) a semi-quantitative analysis that determines the ratio of tracer uptake between the heart (H) and the contralateral half of the lung (CL), referred to as the H/CL ratio [80] **(**Fig. 3**)**.

A number of single-center studies have confirmed the high diagnostic accuracy of ^99m^Tc-DPD, ^99m^Tc-HMDP and ^99m^Tc-PYP, yielding a sensitivity and specificity of > 90% [78, 81–84]. In line, a recent meta-analysis comprising 529 patients has reported a pooled sensitivity and specificity of 92% (95%-CI: 89–95%) and 95% (95%-CI: 77–99%), respectively [85]. Importantly, however, 99mTc phosphates are not specific to ATTR-related amyloid deposits but also exhibit increased focal uptake in a substantial proportion of patients (up to 20%, but not all) with AL amyloidosis. Exclusion of AL amyloidosis by means of proving the absence of monoclonal proteins in serum and urine electrophoresis is therefore mandatory and confers the very high specificity of scintigraphy for the detection of cardiac ATTR-amyloid. A large international collaboration by Gillmore et al. [86] retrospectively evaluated the diagnostic performance of nuclear imaging in over 1200 patients referred for evaluation of suspected cardiac amyloidosis. Disregarding some methodological issues, the authors concluded that the collective findings of a Perugini score ≥ 2 on planar scintigraphy along with the absence of monoclonal gammopathy on serum and urine electrophoresis yield a specificity and positive predictive value of 100% for ATTR cardiac amyloidosis, allowing confirmation of the diagnosis without the need for endomyocardial biopsy. Interestingly, however, the specificity of scans with a Perugini score ≥ 2 was only 91% in the absence of exclusion of monoclonal protein. This underlines the necessity to exclude AL cardiac amyloidosis by immunofixation studies concurrently with bone-tracer imaging.

The main advantages of scintigraphy are its relatively low costs, its established reimbursement in Germany, the wider availability compared to CMR, and the independence of image quality from patient-specific factors. Hence, scintigraphy is a highly valuable diagnostic tool for screening patients with suspected cardiac ATTR amyloidosis and current international guidelines recommend its use early in case of clinical symptoms, ECG, echocardiography, and/or CMR findings suspicious for cardiac amyloidosis [87, 88]. However, as outlined by Kittleson et al. recently [89], the test performance of scintigraphy in populations with lower disease prevalence is unknown and the aforementioned positive predictive value of scintigraphy (with the absence of monoclonal gammopathy) of 100% for ATTR is questioned by some recent reports that illustrate possible causes of false-positive 99mTc-PYP scans [90, 91]. In addition, first larger studies with a direct comparison of scintigraphic ^99m^Tc-DPD images with a CMR-based determination of ECV in the same patients suggest that there is a prognostic significance for the more precise CMR-based ECV [70].

With the recent introduction of therapeutic options, a need for diagnostic modalities allowing for treatment monitoring did arise. Therefore, means of quantifying disease burden become a requirement for future applications of imaging. Planar scintigraphy and SPECT imaging, however, inherently lack the possibility of quantification. By contrast, CMR-based determination of, e.g., the myocardial ECV in patients with proven cardiac amyloidosis may evolve as a valuable tool for estimating the extent of amyloid (including the differentiation of degrees of severity or the monitoring of progression over time) [92]. Alternatively, positron emission tomography (PET) may play an important future role, as it inherently provides quantitative measures of radionuclide uptake, potentially allowing for accurate assessment of the treatment response similar to that established for follow-up of inflammatory myocardial disease (e.g., cardiac sarcoidosis).

#### PET for the diagnosis of cardiac amyloidosis

Due to the limited number of (mostly retrospective) studies with small case numbers, it is not yet possible to make reliable statements on the use of promising positron emission tomography (PET) for the diagnosis of cardiac amyloidosis [93]. In previous PET studies, the following radionuclides have been used: ^11^C-Pittsburgh Compound (PIB), ^18^F-florbetaben, and ^18^F-florbetapir [94–96]. These radionuclides were primarily developed to detect β amyloid plaques in the brains of Alzheimer’s patients and appear to allow detection of both ATTR and AL deposits in the heart. While ^18^F^−^based radionuclides have a relatively long half-life of > 100 min and, therefore, do not necessarily need to be produced on site, PIB has a relatively short half-life and, therefore, needs to be used quickly after production. Dependent on tracer and technique, the average radiation exposure of amyloid-targeted PET ranges from 1.5 to 7 mSv per patient and examination [97, 98]. For these radionuclides, diagnostic sensitivities of 87–100% and specificities of 83–100% for the diagnosis of cardiac amyloidosis have been determined in smaller monocentric studies [93]. Future studies are yet required to assess the potential of PET to differentiate between cardiac ATTR and AL amyloidosis. The strongest potential may, however, be in the quantitative nature, enabling therapy monitoring. Of note, a recent study using PIB-PET confirmed that the quantitative myocardial signal reflects the amount of amyloid deposit and is an independent predictor of adverse outcome in AL [99].

### Endomyocardial biopsy (EMB)

Today, endomyocardial biopsy (EMB) still remains the gold standard for the diagnosis of many non-ischemic heart diseases, such as (viral) myocarditis, cardiac storage diseases, and also for infiltrative or restrictive cardiomyopathies such as cardiac amyloidosis. EMB can therefore result in a therapeutic decision with prognostic relevance in cardiomyopathies, e.g., in inflammatory diseases such as cardiac sarcoidosis or (giant cell) myocarditis. In each individual case, however, the advantages of EMB in terms of diagnosis and therapy implementation must be properly evaluated and weighed against other aspects, such as the sufficient availability of non-invasive information using modern imaging techniques or the potential complication risks of invasive EMB. The specific local conditions and experiences on site as well as the age of the patients should be taken into account.

Larger multi-centre studies have shown that the risk of complications of EMB for both left-ventricular (LV) and right-ventricular (RV) biopsy is low (< 1.0%) in experienced centres with high case numbers, and that serious or permanent complications are rare [100, 101].

Another important limitation of EMB procedures in previous studies was often the so-called “sampling error”: since (viral) myocarditis, for example, has a rather irregular spot-like pattern and the focus of inflammation is often located more subepicardially; the subendocardial EMB sample may be histopathologically negative, even though myocarditis is present. In the case of cardiac amyloidosis, on the other hand, there is a more diffuse distribution of amyloid deposits (particularly in the advanced stage), especially in the basal layers of the LV, which, in most cases, also extends from subendocardial to intramural. In this respect, the risk of a “sampling error” of the subendocardial collection of an EMB in case of cardiac amyloidosis is lower than in case of myocarditis. Collection of at least 5 LV or RV samples from the target region (if possible previously defined by imaging) is recommended for cardiac amyloidosis [102].

**Fig. 4:**
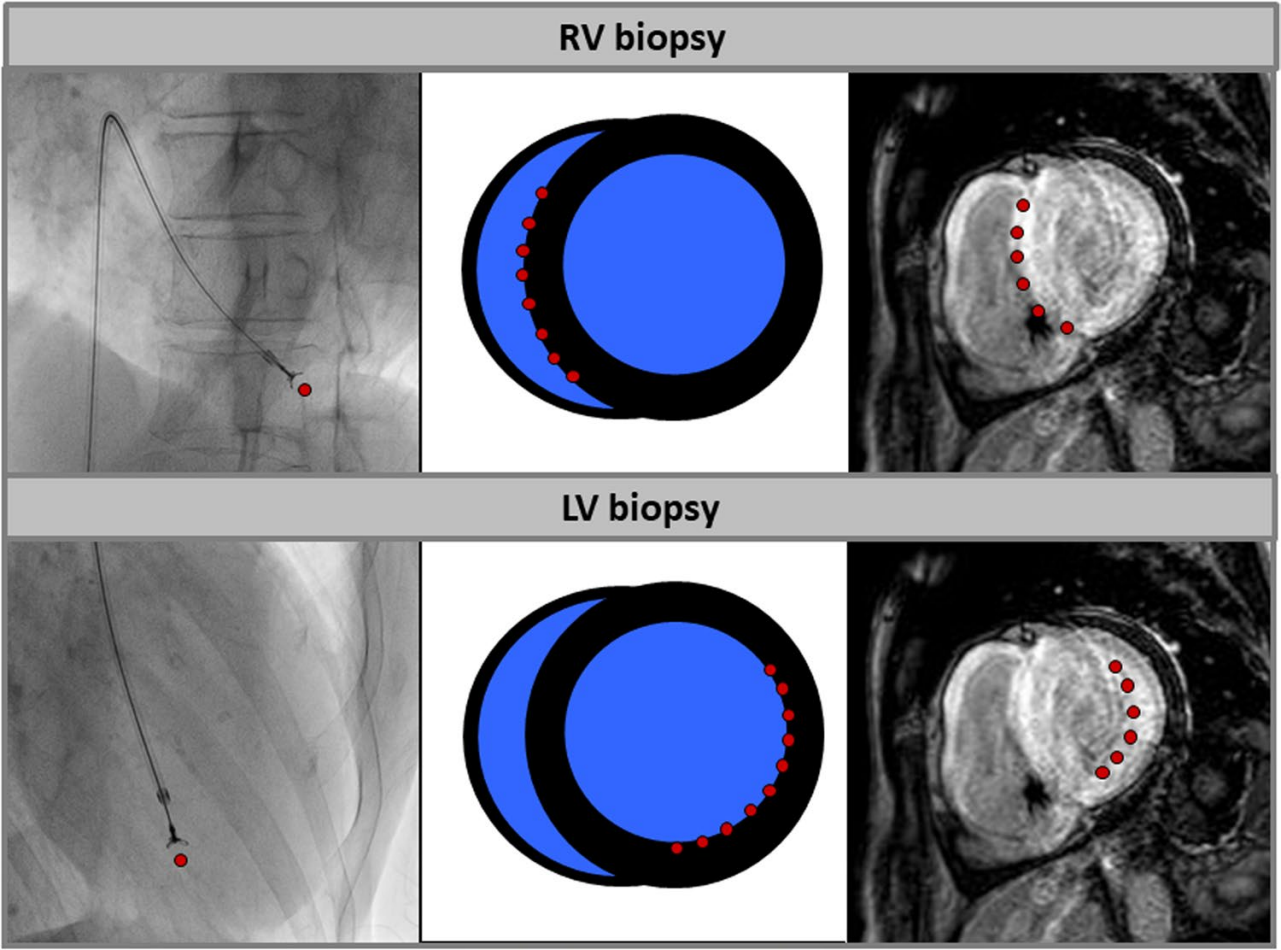
Schematic illustration of the target regions of endomyocardial biopsy of the right (RV) or left ventricle (LV). While in case of RV biopsy, the right-ventricular parts of the septum can be reached very well, an LV biopsy allows sampling of tissue in the area of the left-ventricular lateral or inferolateral wall easily. In advanced cardiac amyloidosis, the risk for a sampling error is quite low for both RV and LV biopsy due to diffuse amyloid deposits

Cardiac amyloidosis often involves both the LV and the RV, and the amyloid deposits are particularly pronounced in the septum as well as in the area of the free LV lateral wall. Since the septum can be reached particularly well with an RV biopsy and the LV lateral wall with an LV biopsy, both RV and LV biopsy are in principle suitable for the bioptic diagnosis of cardiac amyloidosis **(**Fig. 4**)** [103]. In individual cases, the availability of data from previous imaging (e.g., LGE distribution pattern in CMR; orientation of the septum in the transverse plane) is helpful, as this allows to better define in advance the target region for the biopsy as well as the methodological procedure and to reduce potential risks (e.g., unintentional puncture of the free RV wall).

The biopsy-proven extent of amyloidosis is of crucial therapeutic importance, particularly in AL amyloidosis: a bioptically quantified amyloid load of > 20% (of the EMB sample considered) is associated with a lower probability of a therapeutic benefit from consecutive chemotherapy [104]. For both ATTR and AL amyloidosis, there is evidence that a relatively high myocardial amyloid load is associated with a worse prognosis [105]. However, since (a) amyloid deposits in the myocardium can occur both in an irregular nodular fashion and diffusely interstitially and (b) a standardized procedure for the quantification of the amyloid load in EMB samples of cardiac amyloidosis is still lacking, valid and clinically usable data on the significance of the extent of amyloid, oedema, or inflammation in the EMB sample are not yet available.

In the current heart failure guidelines of the ESC, an EMB in patients with progressive heart failure symptoms is primarily recommended if (a) it is necessary to confirm the diagnosis and (b) has direct therapeutic consequences (strength of recommendation IIa/C) [53]. In general, these conditions currently apply in cases of suspected cardiac amyloidosis. Especially with regard to the increasing availability of new drug therapies, bioptic diagnosis including exact differentiation of cardiac amyloidosis (e.g., by immunohistochemical staining) will continue to play a decisive role for the time being.

### Extracardiac biopsy

Extracardiac tissue biopsy is used to confirm the diagnosis of systemic amyloidosis, especially in the case of non-cardiac involvement. The diagnostic certainty of non-cardiac sampling depends on the type of amyloidosis and the tissue examined [27]. Sampling from an affected organ is essential, as the sensitivity of the tissue sample for amyloid detection is highest. In a study with 286 patients with cardiac ATTR amyloidosis, only 73% of the patients showed positive amyloid detection in an extracardiac biopsy [106]. In patients with ATTR amyloidosis, on the other hand, confirmation of the diagnosis using non-cardiac biopsies was only possible in about 30% of cases. AL amyloidosis is most frequently detected in extracardiac biopsies, followed by ATTRv amyloidosis, whereas the sensitivity for the detection of ATTRwt amyloidosis is low in extracardiac biopsy. Therefore, if cardiac amyloidosis is suspected, it is recommended to perform amyloidosis diagnostics on endomyocardial biopsies, which have a significantly higher sensitivity if there is no unequivocal diagnosis made from non-invasive testing. More detailed explanations on extracardiac diagnostics can be found in the attachment (additional material online: Attachment Extracardiac Biopsy).

### Histological examination of biopsies for suspected amyloidosis

**Fig. 5:**
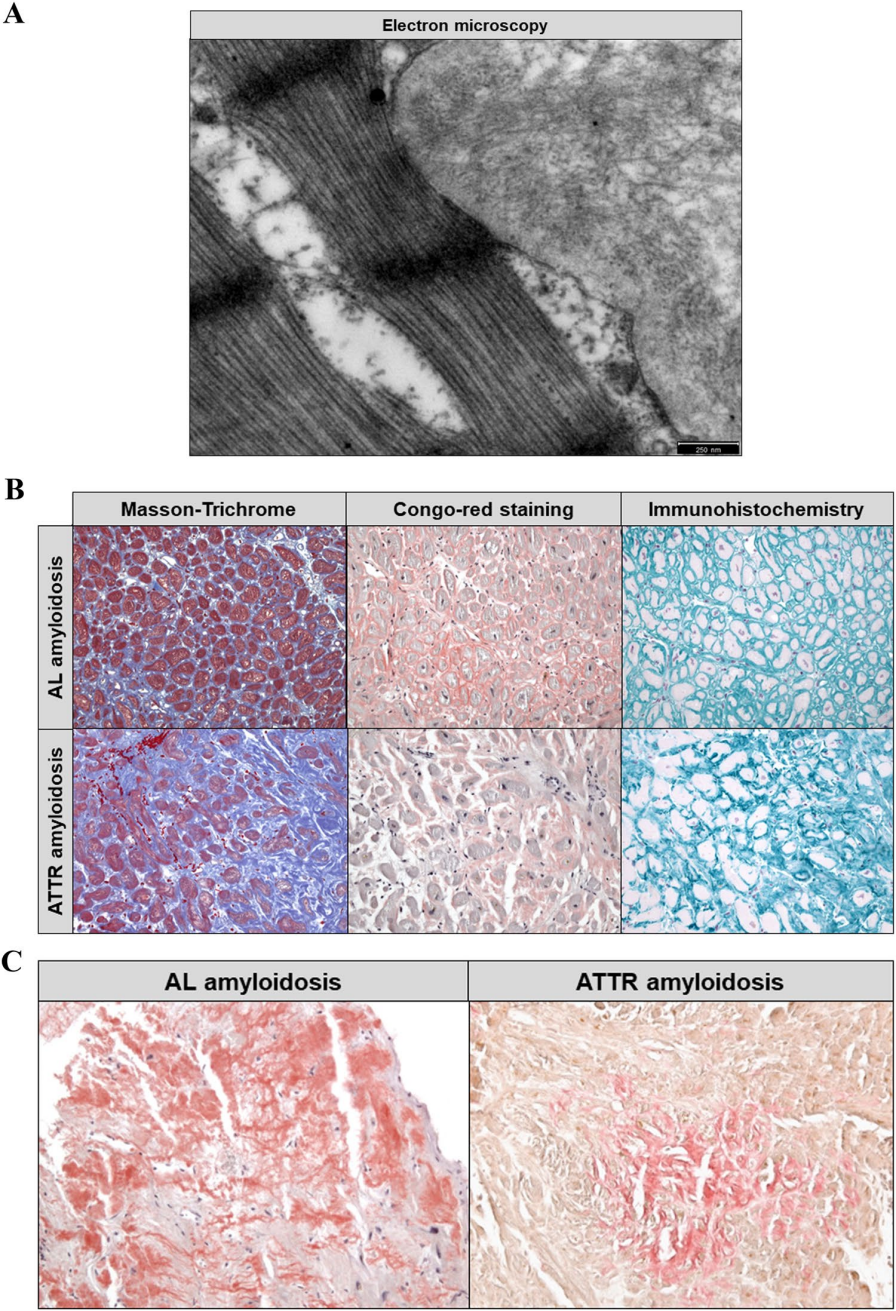
**a** Electron microscopic detection of amyloid protein fibrils (in an endomyocardial tissue sample) that show a characteristic structure of antiparallel beta strands with a diameter of 7–13 nm. **b** Histopathological studies including immunohistochemical stainings allow an accurate classification of the amyloid subtype at an early stage of disease. **c** Typically, in cardiac AL amyloidosis, a rather reticular pattern is found, whereas in ATTR amyloidosis, focal amyloid deposits predominate

All amyloid deposits consist of protein fibrils that have a remarkably similar structure of antiparallel beta strands (more rarely parallel beta strands) with a diameter of 7–13 nm, which can be easily identified by electron microscopy (EM) in the myocardium **(**Fig. 5a**)**. Recently, EM studies revealed that pathological fibrils from brain and heart exhibit morphological homogeneity and that the physiological milieu is the key determinant of amyloid fibril strains [107]. Formalin-fixed and paraffin-embedded (FFPE) tissue samples of the left ventricle or right ventricle are routinely used for the diagnosis of cardiac amyloidosis. Amyloid is detected by Congo red staining, which shows a typical apple-green, but partly also yellow–orange birefringence when viewed under polarized light. The correct Congo red staining is also critical in heart biopsies, as these can easily be overstained and the result is then interpreted as false positive.

Crucial for the therapy is the accurate classification of the amyloid, which can be reliably performed already at an early stage of the disease with high sensitivity by immunohistochemical (IHC) staining in FFPE EMB. Reliable antibodies are used for the detection of ATTR, AL, and AA amyloidosis, which account for more than 99% of cardiac amyloidoses **(**Fig. 5b**)**. Typically, in cardiac AL amyloidosis, a rather reticular/pericellular pattern is found, whereas in ATTR focal amyloid deposits predominate ([104]; Fig. 5c). In some designated laboratories, additional antibodies are available which can be used to identify rare cardiac amyloidoses (e.g., apolipoprotein AI, Lect2, and β2-microglobulin).

For the immunohistochemical differentiation of amyloidoses, it must be taken into account that the incidence of monoclonal gammopathy of unclear significance (MGUS) is high (5.3% in > 70 years old, 7.5% in > 85 years old) [108], 25% of patients with ATTRv or ATTRwt amyloidosis have in parallel MGUS, and 10% of systemic amyloidoses are misdiagnosed with regard to the form of amyloidosis [109].

For the identification of amyloid, there are further stains as thioflavin T or S, but the fluorescence microscopy required for this is rarely available. Furthermore, staining with thioflavin is not entirely specific for amyloid. In recent years, luminescent conjugated polythiophenes (LCP) and oligothiophenes (LCO) have been developed for the histological diagnosis of amyloid, including h-FTAA (“heptamer formyl thiophene acetic acid”). The application of laser dissection mass spectrometry LDMS or mass spectrometry-based quantification by isotope-labeled cell-free products (MS-QBIC) in FFPE EMB might be useful for the classification of systemic amyloidoses.

### Genetic testing for suspected ATTR amyloidosis

ATTR amyloidosis is the most common familial form of amyloidosis and is caused mainly by non-synonymous mutations in the *transthyretin* gene (*TTR*; Chr. 18q12.1; 147 amino acids), which lead to instability of the protein's tetrameric structure. There are now more than 100 *TTR* gene mutations known, of which about 40% are associated with systemic ATTR and 45% ATTRv and neurological manifestation. The transmission of mutations is autosomal dominant, with variable penetrance and clinical manifestation. A familial occurrence of amyloidosis-related disease signs might be indicative of an inherited form.

Non-genetic forms of amyloidosis with cardiac involvement are, for example, the ATTRwt amyloidosis (criterion: exclusion of a *TTR* gene mutation) and ANF-associated amyloidoses (AANF), which manifest primarily as atrial cardiomyopathy. Other hereditary amyloidoses include genes for the apolipoprotein A-2 (APOA2; amyloidosis subform: AApoA2), for the α fibrinogen (FGA), the gelsolin (GSN, “finnish amyloidosis”; amyloidosis subform: AGel), lysozyme (LYZ; ALys), cystatin C (CYS3; ACys), or the “β precursor protein” (Aβ), but do not show any cardiac manifestation.

#### Recommendations for genetic testing in cardiac amyloidosis

- In an index patient with cardiac, but unclassified ATTR amyloidosis (after biopsy examination), a molecular genetic diagnosis for further differentiation (ATTRwt vs. ATTRv) with sequencing of the *TTR* gene should be performed.
- In selected cases, a genetic analysis of the *TTR* gene might be performed if clinical findings and in particular non-invasive imaging (echocardiography, CMR) suggest a strong suspicion of cardiac amyloidosis or otherwise unexplained LV hypertrophy; laboratory tests should have ruled out monoclonal gammopathy.
- In selected cases, an extended genetic diagnosis of further amyloidosis genes (e.g., if AApoA1 is suspected) may also be considered.
- Biologically related first-degree relatives of an index patient with a proven hereditary cardiac amyloidosis should be examined for signs of cardiac amyloidosis regardless of symptoms, and should undergo a mutation cascade screening to determine the mutation carrier status (after respective genetic counselling according to the current national gene diagnostic law).

### Correlations between genotype and cardiac phenotype in ATTRv

A regional accumulation of ATTR amyloidosis is found in Japan, Sweden, and Portugal, particularly of ATTRv with neurological manifestation with a specific and most common *TTR* gene mutation (p.Val50Met). The age peak varies regionally (“early onset” in Portugal and Japan: 30–40 years; “late onset” in Sweden: 50–60 years). In the case of organ involvement, the polyneuropathy dominates (cardiac involvement usually only manifests itself later in life), although the gastrointestinal tract and the eyes may also be affected.

*TTR* gene mutations with primary cardiac involvement include the amino acid exchange mutations p.Val40Ile, p.Thr649Ala, p.Thr80Ala, p.Ile88Leu, p.Leu131Met, and p.Val142Ile (4% for African Americans). In a large study, it was shown that both advancing age of onset of the disease and lower mean arterial blood pressure were independently associated with a higher mortality compared to a specific, non-synonymous gene mutation [35].

A significant prognostic difference exists between AL and ATTR patients. In addition, smaller studies indicate that, within the ATTR group, certain forms of ATTRv (e.g., p.Thr80Ala or p.Val142Ile) have been associated with a particularly poor prognosis [36, 110].

### Diagnostic pathway for cardiac amyloidosis

**Fig. 6:**
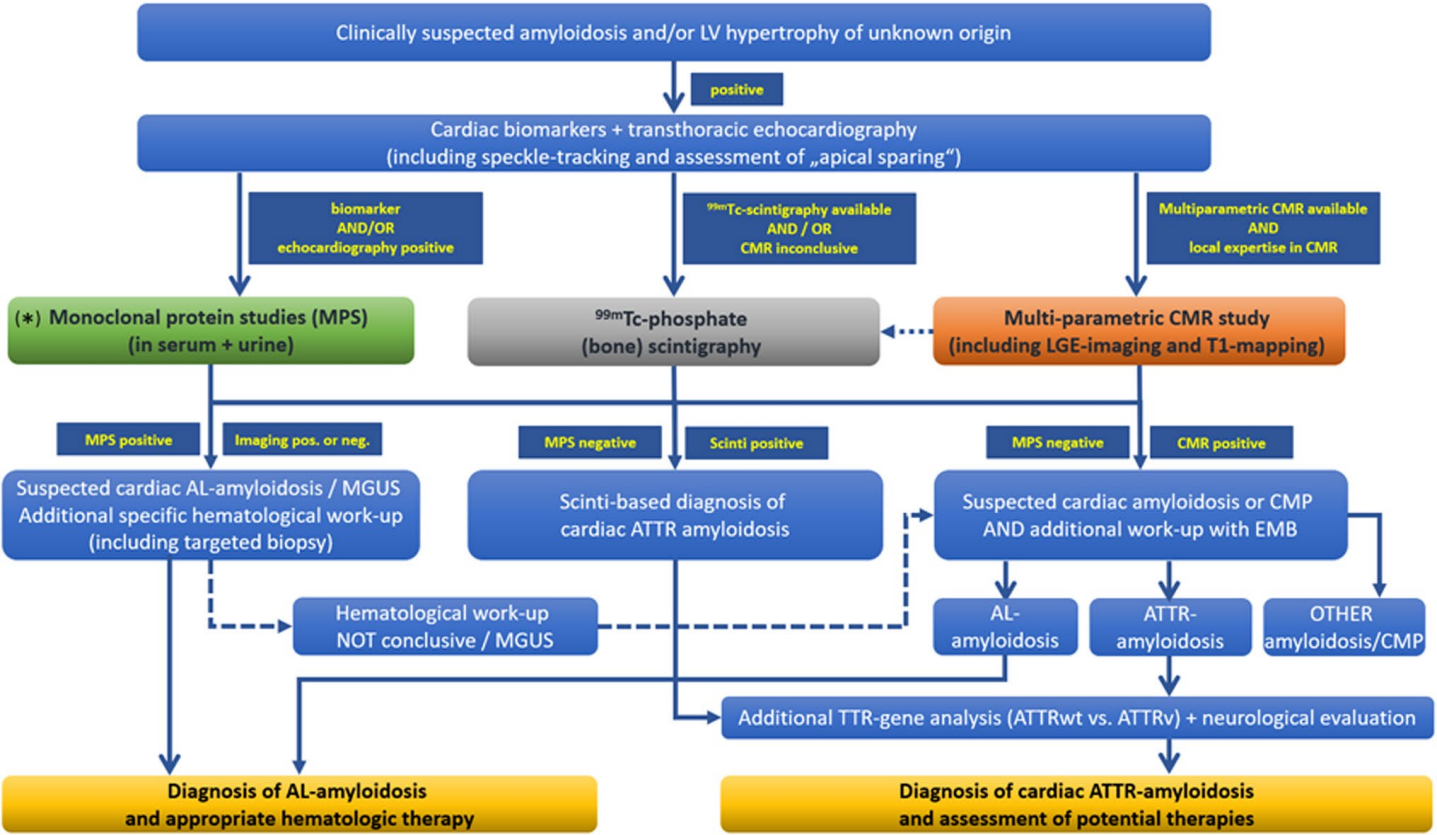
Recommended path for the diagnostic work-up of suspected cardiac amyloidosis. The main objective of this diagnostic path is to detect the presence of cardiac amyloidosis as reliably and early as possible, to characterize the extent of cardiac amyloidosis as precisely as possible, to reliably identify the underlying type of amyloidosis, and to subsequently enable targeted treatment (including the possibility of monitoring the success of therapy). * = *If cardiac amyloidosis is suspected, further clarification by means of free light chains and immunofixation for the determination of light chains is recommended promptly and without waiting for additional imaging results*

In view of the data available to date on the various non-invasive imaging procedures and invasive endomyocardial biopsy, we recommend—in addition to the laboratory tests described above—the diagnostic path outlined in Fig. 6. The main objective of this diagnostic path is (i) to detect the presence of cardiac amyloidosis as reliably and early as possible, (ii) to characterize the extent of cardiac amyloidosis as precisely as possible, (iii) to reliably identify the underlying type of amyloidosis, and (iv) to subsequently enable targeted treatment (including the possibility of monitoring the success of therapy). The implementation of this diagnostic path is of course dependent on the available options on site as well as local expertise and experience.

If cardiac amyloidosis is suspected, further clarification by means of free light chains and immunofixation (from serum and 24 h urine) for the determination of light chains as well as CMR and/or ^99m^Tc-phosphate scintigraphy is recommended promptly—if possible in a specialized amyloidosis centre. Since AL amyloidosis progresses rapidly and the patient's prognosis depends on the extent of cardiac involvement and immediate initiation of therapy, an immunofixation consisting of serum and 24-h urine collection should be initiated immediately on site. This will allow for a timely presentation to a hematology service in case of detection of a monoclonal gammopathy.

Importantly, the decision whether to perform a multi-parametric CMR study and/or a ^99m^Tc-phosphate scintigraphy for further non-invasive work-up of suspected cardiac amyloidosis depends (amongst others) on (a) local availability and (b) respective imaging expertise. Hence, the diagnostic path outlined in Fig. 6 needs to consider local peculiarities. The same is true regarding invasive sampling of EMBs.

It remains to be seen to what extent non-invasive imaging procedures will not only allow for the diagnosis of cardiac amyloidosis in the future, but also a reliable differentiation of different types of amyloidosis, thereby reducing the need for invasive EMB. In individual cases, an invasive EMB can be dispensed (especially in very old patients with severe frailty precluding invasive diagnostics) if (a) unequivocal echocardiographic, CMR, or scintigraphic findings of cardiac amyloidosis are available, (b) the presence of monoclonal bands has been ruled out, and/or c) a specific drug therapy is not considered. In addition, even in younger patients (< 50 years) with a family history and/or a symptom complex that clearly indicates systemic ATTRv amyloidosis, an EMB can be omitted for the time being and a genetic examination ± extracardiac biopsy can be performed. Importantly, future studies need to further clarify whether an imaging-based diagnosis of cardiac amyloidosis—without a histopathological proof of amyloidosis—is appropriate and sufficient prior to initiating specific, however expensive, therapies (e.g., in case of ATTRwt).

However, due to the higher significance, EMB is still primarily recommended if cardiac amyloidosis is suspected. If an EMB is not technically possible, not desired by the patient, or not purposeful, an extracardiac biopsy (especially FAB) can be considered—despite a significantly lower sensitivity.

In asymptomatic ATTRv mutation carriers, a comprehensive screening for cardiac manifestation comprising ECG, serum biomarkers, and echocardiography is suggested to be performed 10 years prior to the predicted age of onset and annually thereafter [111]. The initial assessment may also include CMR or ^99m^Tc-phosphate scintigraphy—depending on the underlying TTR gene mutation.

## Treatment of cardiac amyloidosis

### General treatment recommendations

In principle, the same general treatment recommendations apply to patients with symptomatic cardiac AL and ATTR amyloidosis as for patients with heart failure (HFrEF or HFpEF) due to other causes [53]. In amyloidosis patients, however, it should be noted that even low doses of β lockers or ACE inhibitors can lead to symptomatic hypotension and are often not well tolerated. Since patients with cardiac amyloidosis also have a limited coronary perfusion reserve, a β-blocker therapy with consecutive reduction of the heart rate can lead to a drop in cardiac output with clinical deterioration of the state of compensation. Therefore, heart failure therapy in patients with cardiac amyloidosis is primarily based on the correct titration of diuretics.

There are currently insufficient data on the use of sacubitril/valsartan in patients with cardiac amyloidosis. A possible acceleration of amyloid deposition by sacubitril/valsartan was discussed.

Older case studies indicate that calcium channel blockers (in particular nondihydropyridine calcium channel blockers) or digitalis preparations should be used with restraint or caution in patients with cardiac amyloidosis, as diastolic dysfunction may further deteriorate and heart block may occur [112].

### Specific drug therapies for AL amyloidosis

The timely treatment of underlying plasma cell dyscrasia or clonal B-cell disease is the main focus of AL amyloidosis therapy. The treatment regimes used are similar to those used for multiple myeloma. There is no specific therapy for cardiac involvement, but the degree of cardiac involvement often limits the choice of chemotherapy. The choice of therapy is based on dedicated risk stratification and includes criteria such as age, Karnofsky index, number of organs involved, and extent of organ involvement (kidney function, cardiac stage according to Mayo staging system, and NYHA stage) [113]. If the patients are suitable, a high-dose melphalan therapy followed by autologous stem cell transplantation is aimed for [114]. For patients in advanced stages, conventional therapies such as melphalan/dexamethasone or various combination therapies with the proteasome inhibitor bortezomib (or ixazomib) or immunomodulators are used [115, 116].

The duration of therapy depends on the response according to established international response criteria [117]; therefore, close monitoring of the activity parameters is necessary every 2–3 cycles. If the patient does not respond to primary therapy, an immediate switch to second-line therapy is mandatory. The treatment scheme should be determined in a specialised centre (also to allow inclusion in studies). As a non-drug therapy option, heart transplantation may be considered in selected cases to enable patients to receive high-dose chemotherapy and autologous stem cell transplant afterwards.

### Specific drug therapies for cardiac ATTR amyloidosis

In the field of ATTRv amyloidosis, several different drug-based treatment strategies have been developed in recent years. With the aim of slowing down the progress of the disease, drugs that either stabilize the transthyretin tetramer or suppress the synthesis of the protein in the liver were tested in clinical trials. In Europe, three drugs are currently approved for the treatment of ATTRv polyneuropathy, and one drug was approved for therapy of amyloid cardiomyopathy (hereditary and wild-type) recently.

Since 2011, the TTR stabilizer Tafamidis-meglumin (Vyndaqel^®^) has been approved in Europe for the treatment of stage 1 amyloid polyneuropathy. In the first quarter of 2020, the European Medicines Agency (EMA) approved Tafamidis for treatment of amyloid cardiomyopathy based on the data from the recently published phase III clinical trial (ATTR-ACT). In this study, the safety and efficacy of Tafamidis (20 mg vs. 80 mg vs. placebo) was evaluated over 30 months in a cohort of patients with amyloid cardiomyopathy (106 ATTRv and 335 ATTRwt patients) [118]. Compared to the placebo group, a significant reduction in mortality was observed in all subgroups. The second endpoint “reduction of hospitalizations due to cardiac decompensation” was achieved in all subgroups—except NYHA III patients. There were also positive effects on the walking distance in the 6-min walking test and on the quality of life in the KCCQ-OS questionnaire. The approved dosage for treatment of amyloid cardiomyopathy is Tafamidis 61 mg, the bioequivalent of 80 mg Tafamidis-meglumine. Serious side effects are not expected under treatment with tafamidis [119]. The study data suggest that starting therapy as early as possible has a positive effect on patient outcome. Hence, treatment should be initiated as early as possible—if all diagnostic and study criteria are met. For patients in advanced stages (NYHA III), the decision to start Tafamidis therapy should be made after careful individual assessment and in consideration of comorbidities, general condition, and inclusion/exclusion criteria of the ATTR-ACT study. No data are available for heart failure NYHA class IV.

Since 2018, two TTR-mRNA interfering drugs, the RNA interference (RNAi) drug patisiran (Onpattro^®^) [120] and the antisense oligonucleotide inotersen (Tegsedi^®^) [121], have been approved in Europe, both for the treatment of ATTRv polyneuropathy in stages I–II. In both pivotal studies, the primary neurological endpoints were met. Since patients with mixed phenotype were also included, first subgroup analyses for patients with cardiac involvement exist. Patisiran showed a significant reduction of LV wall thickness and NT-proBNP levels in a subgroup of ATTRv patients [122]. However, since in this study, cardiac amyloidosis was confirmed neither bioptically nor by means of CMR-based or scintigraphic methods, these initial results should be interpreted with caution.

Despite slight differences between the study cohorts, it can be assumed that, based on the study results to date, both patisiran and inotersen are equally effective and well tolerated. Since the use of these drugs is currently only possible for neurological manifestation, an interdisciplinary approach is obligatory. Positive effects on the progression of cardiomyopathy should be expected for both drugs on the basis of the previous subgroup analyses. Reliable study data regarding the prognostic relevance in patients with cardiac amyloidosis are not yet available, but two clinical trials were recently initiated to evaluate the safety and efficacy of RNAi drugs for the treatment of amyloid cardiomyopathy (NCT0413671 and NCT04153149).

Furthermore, there is some evidence that the polyphenol epigallocatechin gallate (EGCG) may have a positive effect on the clinical course of both types of cardiac ATTR amyloidosis [123], although peer-reviewed results of the corresponding prospective randomized studies are not yet available. It can be taken as a dietary supplement and a dose of 800–1200 mg EGCG with added vitamin C is recommended. Positive effects have also been described for the combination of doxycycline and Urso-falk^®^ [124], which can be used as off-label therapy.

Obviously, addressing cost-effectiveness issues for novel ATTR therapies is not within the scope of this position statement. However, we encourage cost-effectiveness analyses such as performed recently by Kazi et al. [125] for use of Tafamidis (in ATTR cardiomyopathy)—illustrating that a price reduction of more than 90% is required to make Tafamidis cost-effective at $100,000 per quality-adjusted life-year (QALE). Considering the growing number of patients with cardiac amyloidosis (in particular with ATTRwt), cost-effectiveness issues need to be carefully considered in the future regarding specific, however expensive, drugs addressing amyloid cardiomyopathy.

### Specific drug therapies for AA amyloidosis

Due to the diversity of the various triggering underlying diseases, there is no generally valid treatment strategy for AA amyloidosis. The fundamental and common goal of all therapies is the inhibition of the synthesis of the precursor protein SAA [126]. Depending on the underlying disease, various drugs are used, such as anti-infectives for infectious-inflammatory diseases, immunosuppressants for autoimmune diseases, and chemotherapy for malignant diseases. The target of newer therapies is the precursor protein itself; initial results are available, for example, for the monoclonal anti-IL-6 antibody tocilizumab. There is no specific drug therapy for heart involvement.

### Liver transplantation in ATTR amyloidosis

Transthyretin, the protein underlying ATTR amyloidosis, is produced almost exclusively in the liver. Hence, liver transplantation is a possible treatment option for patients with ATTRv [127]. The genuine liver, which produces the variant form of transthyretin, is replaced by a donor liver that synthesizes the wild-type form of transthyretin. After transplantation, there is no further production of variant transthyretin, and the progression of neurological as well as of cardiac symptoms should be stopped [128, 129]. The liver of a mutation carrier which is healthy in terms of detoxification and synthesis performance can be passed on to a patient with another liver disease on the transplant waiting list in the sense of a domino liver transplant. However, it should be noted that the organ recipient will also develop ATTRv amyloidosis in the long term.

According to the most recent figures of the worldwide “Familial World Transplant Registry” (http://fapwtr.org), approximately 120 patients with ATTRv receive a liver transplant per year, and by 2017, a total of 68 ATTRv patients received liver transplants in Germany. This therapeutic option is associated with a 10-year survival rate of 21–85% depending on the underlying mutation. Independent factors for a favourable outcome are a higher body mass index, an early onset of the disease (age < 50 years), a short disease duration, and the presence of a Val30Met mutation [128].

In some ATTRv patients, especially those with other than p.Val50Met mutations, further disease progression was observed even after liver transplantation [129]. Mass spectrometric analysis of tissue from affected patients showed a deposition of wild-type transthyretin on existing ATTRv amyloid. Therefore, liver transplantation should be performed at the earliest possible disease stage with a still low level of amyloid deposition. On the other hand, it seems reasonable to treat ATTRv patients with transthyretin-stabilizing drugs even after liver transplantation. In patients with advanced cardiac ATTRv amyloidosis, a combined or sequential transplantation of liver and heart can be considered [130]. Of note, a paradoxically accelerated amyloid deposition in the myocardium following a liver transplantation can also occur [131].

Nevertheless, with the approval of new gene-silencing drugs, which eliminate variant transthyretin from the blood [132], analogous to liver transplantation, the indication for liver transplantation must be critically reviewed, especially in patients with other than Val30Met mutations [128]. However, no comparative studies are available to date.

ATTRwt amyloidosis, on the other hand, is based on the deposition of non-mutated wild-type transthyretin, which would be produced in the same way by the donor organ even after a liver transplant. Liver transplantation is therefore not a therapeutic option for this type of amyloidosis.

### Device therapy for cardiac amyloidosis

The clinical decision for or against a device therapy (e.g., pacemaker or ICD) in a patient with cardiac amyloidosis is not easy, since there is a lack of robust evidence-based studies and usually a decision has to be made on a case-by-case basis, carefully considering the individual constellation of findings. On one hand, the type of amyloidosis, the stage of disease, and individual prognosis must be taken into account. In principle, device therapy is only considered if a median life expectancy of at least 1 year is to be expected—which should be carefully considered in advanced amyloidosis with cardiac involvement. On the other hand, a comprehensive cardiac evaluation should have been carried out and it should also be taken into account whether special or specific therapies are pending (e.g., chemotherapy for AL amyloidosis).

Common causes of death in patients with cardiac amyloidosis are a (rapidly) progressive heart failure and sudden cardiac death. While some studies have documented a high incidence of ventricular arrhythmias and have even shown a prognostic significance for the occurrence of ventricular couplets or nsVTs [133, 134], other studies indicate a greater significance of bradycardic arrhythmias (especially higher grade AV blockages or electromechanical decoupling) as triggering factors for decompensation or sudden cardiac death [135]. A causally obvious benefit of pacemaker or ICD therapy (e.g., in the form of a reduction in mortality or SCD risk) has so far not been observed in larger studies [136, 137]. However, it needs to be considered that in individual cases and smaller studies, convincing evidence of the benefit of ICD therapy in patients with cardiac amyloidosis has been provided [134, 138, 139].

Due to those limited and sometimes contradictory data, the recommendations of the European and American societies have also been divergent in the past. While the ESC guidelines from 2015 only recommended secondary prophylactic ICD implantation in patients with amyloidosis and documented persistent ventricular arrhythmia (strength of recommendation IIa/C) [140], the American guidelines from 2013 also mentioned primary prophylactic indications [141] (including amyloidosis patients with limited LV function (LVEF < 50%) and unclear syncope). However, in the American guidelines updated in 2017, these recommendations were withdrawn; instead, only an individual decision is recommended [142]. Recently published recommendations from the Heart Rhythm Society (HRS) suggest ICD implantation in patients with AL amyloidosis in case of nsVTs and an expected survival of > 1 year (IIb/C)—but do not separately address patients with ATTR amyloidosis [143].

Meanwhile, some laboratory parameters (e.g., troponin or NT-proBNP) and imaging parameters (e.g., ECV extent in CMR imaging) exist, which allow better risk stratification with regard to mortality and sudden cardiac death (SCD). In view of the limited data available to date and the anticipated longer life expectancy due to new drug therapy options, the authors of this position statement recommend in addition to the above-mentioned ESC recommendations (a) also a careful decision on a case-by-case basis and (b) a rather generous (primary prophylactic) indication for ICD implantation in patients with an increased mortality risk according to serum or imaging parameters and/or documented nsVTs if the life expectancy is > 1 year.

For cardiac resynchronization therapy (CRT), there are no reliable data for patients with cardiac amyloidosis. In a few individual cases, the benefits of a CRT have also been shown for these patients, so that, in principle, a CRT can also be considered for amyloidosis patients and the current recommendations for the CRT indication should be applied [144].

## Clinical follow-up in cardiac amyloidosis

In patients with amyloidosis, serial comprehensive follow-up examinations should be performed including the assessment of all involved organs/organ systems and, in AL amyloidosis, of the hematopoietic system. In AL amyloidosis, the hematologic response often precedes organ response and guides the way regarding optimal hematologic treatment.

For the assessment of the clinical course of cardiac amyloidosis and the respective treatment effect, primarily laboratory biomarkers such as NT-proBNP or troponin T (or -I) as well as non-invasive imaging parameters are available [113, 145].

Even though (a) the existing data for serum biomarkers such as NT-proBNP or troponin T (or I) are much more comprehensive than for non-invasive imaging parameters (e.g., CMR parameters) [146] and (b) some staging procedures already take into account the level of NT-proBNP or troponin T (or I) levels [37], a diagnostic “superiority” of these serum parameters in direct comparison to new imaging parameters has not yet been proven. On the contrary, initial CMR results indicate that a more precise risk stratification, for example, with the T1 mapping procedure and the determination of the ECV value, is possible [70]. Simply measuring the LV wall thickness or the LVEF, on the other hand, is insufficient or only of minor importance with regard to the assessment of the success of treatment and to risk stratification of cardiac amyloidosis. For this reason, we currently recommend the relatively comprehensive follow-up mentioned in Table 3 for patients with cardiac amyloidosis, depending on the respective constellation. Further research on this important aspect is urgently indicated.

**Table 3:** Follow-up strategy for cardiac amyloidosis

	AL amyloidosis*	ATTR amyloidosis
During specific drug therapy (possibly also “off-label use”)	**Every 3 months (or after every 2 further therapy cycles):** NT-proBNP^a^ Troponin T or I Treatment success with a drop of > 30% and indication of treatment failure if increase of > 30% compared to the respective previous value **Every 6 months:** Resting ECG + Holter ECG Transthoracic echocardiography including strain measurements If available: CMR including LGE and T1 mapping	**Every 3–6 months:** NT-proBNP^a^ Troponin T or I Assessment of treatment success depends on the respective drug^b^ **Every 12 months:** Resting ECG + Holter ECG Transthoracic echocardiography including strain measurements If available: CMR including LGE and T1 mapping
After remission or in stable condition without specific therapy	**Every 6 months:** Resting ECG NT-proBNP^a^ Troponin T or I Transthoracic echocardiography including strain measurements **Every 12 months:** Holter ECG Additional CMR including LGE and T1 mapping in case of suspected disease progression due to serum biomarkers and/or echocardiographic findings	**Every 6 months:** Resting ECG NT-proBNP^a^ Troponin T or I Transthoracic echocardiography including strain measurements **Every 12 months:** Holter ECG **Every 12 to 24 months:** Additional CMR including LGE and T1 mapping in case of suspected disease progression due to serum biomarkers and/or echocardiographic findings

*In addition to the criteria regarding cardiac disease response, hematologic response criteria should be measured every 3 months

^a^Measuring of NT-proBNP should be performed in patients in a (re)compensated state (and with a time delay from any cortisone administration)

^b^For Tafamidis, a lack of a further increase in NT-proBNP under therapy has already been interpreted as a therapeutic success, while, for example, under Patisiran, a drop of > 30% to the respective previous value was described after 9 and 18 months [88, 92]

## Summary

Amyloidosis can affect both sexes and individuals of any age. As a multi-organ disease presenting with a variety of rather unspecific symptoms of different severity, the diagnosis of amyloidosis often is delayed, and the number of unreported cases is probably high. Therefore, interdisciplinary cooperation between neurology, hematology, gastroenterology, pathology, and cardiology (and other specialist groups as needed) is an indispensable prerequisite for successful care of the individual patient.

In principle, amyloid can infiltrate all structures of the heart and affect not only the ventricular and atrial walls but also the conduction system, the heart valves, and the coronaries. Therefore, the clinical spectrum of cardiovascular involvement is wide and ranges from asymptomatic courses of dizziness and syncope to the development of restrictive cardiomyopathy and progressive terminal heart failure.

However, the different types of amyloid show different organ tropism typical for the respective disease. Systemic forms of amyloidosis, which affect the heart, are mainly the light chain (AL) and ATTR amyloidoses, resulting from the deposition of misfolded transthyretin (either as the wild-type [ATTRwt] or mutated [ATTRv] form).

In addition to specific cardiac biomarkers, modern non-invasive imaging techniques such as CMR or ^99m^Tc-phosphate scintigraphy are available today, which complement conventional echocardiography and allow not only for the diagnosis of cardiac amyloidosis but also an exact determination of the degree of its manifestation. As an invasive diagnostic procedure, endomyocardial biopsy continues to play a central role in the histopathological confirmation and subtyping of cardiac amyloidosis.

The main objective of the diagnostic path outlined in this position statement is to detect the presence of cardiac amyloidosis as reliably and early as possible, to precisely characterize the extent of cardiac amyloidosis, and subsequently to enable targeted treatment (including the possibility of monitoring the success of therapy)—in consideration of local experience and expertise.

Finally, the targeted treatment of cardiac amyloidosis is increasingly in the focus of clinical trials, and in addition to a label extension for already available substances, the approval of additional newer drugs is expected in the near future.

## Supplementary Information

Below is the link to the electronic supplementary material.

Supplementary file1 (PDF 26 KB)
