# Supplementary material for: Diagnosis and treatment of cardiac amyloidosis: position statement of the German Cardiac Society (DGK)
Source: Clin Res Cardiol. 2021 Jan 18;110(4):479–506. doi: 10.1007/s00392-020-01799-3 (PMC8055575; doi:10.1007/s00392-020-01799-3)
Supplement: Supplementary file 1 — Supplementary file1 (PDF 26 KB) [file 392_2020_1799_MOESM1_ESM.pdf]

Supplement: Extracardiac Biopsy

Depending on the patient's clinical findings, the bone marrow, subcutaneous fatty tissue, salivary glands, the gastrointestinal tract or the affected nerve can be considered for extracardiac biopsy sampling. The biopsy of the sural nerve, which can be used to clarify an amyloid-induced peripheral polyneuropathy, is associated with a high sampling error because the amyloid deposits do not extend over the entire length of the nerve.

Particularly in the early diagnosis of AL and AA amyloidosis, when clinical symptoms are absent, diagnosis can only be confirmed histologically. In this case, screening by means of a fat aspiration biopsy (FAB) is recommended, and in the case of a negative result after a re-biopsy, a biopsy of the symptomatic organ should be done. In principle, FAB is a safe and simple procedure and can be performed subcutaneously using a disposable syringe and usually without local anaesthesia. The advantage of FAB is its very low risk of complications, e.g. in comparison to a liver or kidney biopsy. The specificity of FAB for systemic amyloidosis is very high at 93-100%, but the sensitivity is reported in the literature to vary widely between 14%-90% depending on the form of amyloidosis and on the amount of biopsy material or the thickness of the tissue sections (>5 µm recommended) (ref). Based on 600 patients with cardiac amyloidosis, a sensitivity of 84% for systemic AL amyloidosis could be shown for the FAB[88]. Among patients with ATTR amyloidosis, sensitivity of FAB was significantly lower for the mATTR form at 45% and even lower for the wtATTR form at 15%. Certain forms of amyloid, e.g. A$ß_2$M amyloid, cannot be detected with the FAB.

Kommentiert [AY1]: Ref. korrigieren: Quarta et al, Eur Heart J 2017;38:1905.

All forms of amyloidosis can affect the kidney and result in glomerular, tubulointerstitial and vascular deposits, which can be detected in a kidney biopsy. Systemic AA or AL amyloidoses often lead to kidney involvement. The sensitivity of renal biopsy is over 80% in AL amyloidosis with renal involvement. In patients with AL amyloidosis, a bone marrow biopsy should also be performed if the tissue biopsy is positive and light chains are detected, in order to diagnose or exclude plasma cell dyscrasia such as monoclonal gammopathy or multiple myeloma in order to differentiate between localized and systemic forms.

In several forms of amyloidosis, the gastrointestinal tract (GIT) can be affected at different anatomical sections. In gastrointestinal biopsies, AL amyloidosis is most frequently detected, followed by ATTR amyloidosis. The prevalence of AL(lambda) as well as ATTR amyloidosis increases with sampling from the proximal to the distal GIT; in AL(kappa) amyloidosis it is the other way round. Since, especially in patients with ATTR amyloidosis, the amyloid is mainly found in the submucosa and less frequently in the mucosa, in gastrointestinal biopsy it is recommended to collect tissue from the submucosa with vascular parts in order to avoid sampling error. If submucosa is missing in the biopsy, up to 60% of results may be false negative. Therefore, in case of AL(kappa) or AA amyloidosis a biopsy from the upper GIT should be performed. In case of suspicion of AL(lambda) and ATTR amyloidosis colorectal

biopsies of the submucosa are recommended. The sensitivity of the rectal biopsy for the detection of amyloidosis is reported to be 69-97%.

The biopsy of the sublingual glands has been described with a sensitivity of 91% for the detection of mATTR amyloidosis in case of an existing TTRVal30Met mutation; however, the method is only established in a few countries.
